# Subspecies-level genome comparison of *Lactobacillus delbrueckii*

**DOI:** 10.1038/s41598-023-29404-3

**Published:** 2023-02-23

**Authors:** Min-gyung Baek, Kwan Woo Kim, Hana Yi

**Affiliations:** 1 Interdisciplinary Program in Precision Public Health, Korea University, Seoul, Korea; 2 Department of Public Health Sciences, Korea University, Seoul, Korea; 3 School of Biosystems and Biomedical Sciences, Korea University, Seoul, Korea

**Keywords:** Evolution, Bacterial genomics

## Abstract

*Lactobacillus delbrueckii* comprises six subspecies, *L. delbrueckii* subsp. *bulgaricus*, *L. delbrueckii* subsp. *lactis*, *L. delbrueckii* subsp. *jakobsenii*, *L. delbrueckii* subsp. *delbrueckii*, *L. delbrueckii* subsp. *sunkii*, and *L. delbrueckii* subsp. *indicus*. We investigated the evolution of the six subspecies of *L. delbrueckii* using comparative genomics. While the defining feature of the species was the gene number increment driven by mobile elements and gene fragmentation, the repertoire of subspecies-specific gene gains and losses differed among the six subspecies. The horizontal gene transfer analyses indicated that frequent gene transfers between different subspecies had occurred when the six subspecies first diverged from the common ancestor, but recent gene exchange was confined to a subspecies implying independent evolution of the six subspecies. The subspecies *bulgaricus* is a homogeneous group that diverged from the other subspecies a long time ago and underwent convergent evolution. The subspecies *lactis*, *jakobsenii*, *delbrueckii*, and *sunkii* were more closely related to each other than to other subspecies. The four subspecies commonly show increasing genetic variability with increasing genome size. However, the four subspecies were distinguished by specific gene contents. The subspecies *indicus* forms a branch distant from the other subspecies and shows an independent evolutionary trend. These results could explain the differences in the habitat and nutritional requirements of the subspecies of *L. delbrueckii*.

## Introduction

The *Lactobacillus* genus consists of bacteria that can inhibit the growth of competitors by removing their carbon source and by accumulating organic acids through rapid fermentation. It is among the most economically important genus that has been utilized throughout human history, and it is currently commonly used in industry. Most probiotic products include *Lactobacillaceae,* owing to its proven safety and health benefits. However, the high interspecific 16S rRNA sequence similarity in *Lactobacillus* makes it difficult to distinguish between different species. Therefore, subspecies in this genus have been identified using multilocus sequence typing, which uses multiple genes (*recG, hsp60, recA, pyrG, gyrG, fusA*, and *ileS*) simultaneously^1^. The old *Lactobacillus* genus was recently reclassified and split into 25 new genera. Only 42 species remain in the newly defined *Lactobacillus* genus at the time of reclassification, including *L. delbrueckii*^2^.

*Lactobacillus delbrueckii* was originally described as ‘*Bacillus delbrueckii* ‘ by Leichmann in 1896^3^ and reclassified as *Lactobacillus fermentum* var. *delbrucki* by Beijerinck in 1901^4^. The Judicial Commission of the ICSP decided that the name *Lactobacillus delbrueckii* Beijerinck 1901 shall be held to be validly published by Beijerinck as a species name^5^. Because the original type strain of the species had been lost, and the Judicial Commission indicated in Opinion 38 that its type strain shall be the neotype strain ATCC 9649^T^ = NCDO 213^T^.

The 16S rRNA sequence similarity between its six subspecies is 99.21–99.54%, and *L. delbrueckii* subsp. *delbrueckii* DSM20074^T^ is the type subspecies. Of these six subspecies, most commercial applications of *L. delbrueckii* utilize two key subspecies*,* namely *L. delbrueckii* subsp. *bulgaricus* and *L. delbrueckii* subsp. *lactis*. *Lactobacillus delbrueckii* subsp. *bulgaricus* is used alongside *Streptococcus thermophilus* in the commercial production of yogurt and cheese^6^*. Lactobacillus delbrueckii* subsp. *lactis* is used in the production of cheeses such as mozzarella and parmesan^6^. Because of their industrial importance, the genetic backgrounds of these two subspecies, which account for their differential metabolic functions, have been identified using genomic analyses^7–9^. These two subspecies can be clearly distinguished by the genes involved in carbohydrate metabolism. Although the carbohydrate metabolism of *L. delbrueckii* subsp. *bulgaricus* is mainly concentrated to lactose fermentation and a few additional carbohydrates, *L. delbrueckii* subsp. *lactis* is able to ferment various sugar types of plant origin (such as maltose, mannose, saccharose, and trehalose)^7^.

The subspecies of *L. delbrueckii* subspecies differ with regards to whether they are lactose-negative or lactose-positive. *Lactobacillus delbrueckii* subsp. *delbrueckii*^10^, *L. delbrueckii* subsp. *sunkii*^11^, and *L. delbrueckii* subsp. *jakobsenii*^12^ which were not isolated from dairy products, are lactose-negative; *L. delbrueckii* subsp. *bulgaricus*^10^*, L. delbrueckii* subsp. *indicus*^13^*,* and *L. delbrueckii* subsp. *lactis*^10^, which were isolated from dairy products, are lactose-positive. Besides the dairy and non-dairy fermenting environments, recent metagenomics studies have revealed that *L. delbrueckii* are inhabiting the intestine of human^14^ and animals^15^. Because of the different habitat and nutritional requirements of these six subspecies, they are expected to have different genetic backgrounds that allow them to adapt to differing environments. Although previous research has been based solely on the genomic analyses of *L. delbrueckii* subsp. *bulgaricus* and *L. delbrueckii* subsp. *lactis*, the addition of the other four subspecies to the analyses is expected to contribute to the determination of the evolution of *L. delbrueckii*.

This study aimed to determine the characteristics that can be used to independently define the six *L. delbrueckii* subspecies and to understand the evolutionary trends in these subspecies based on the analysis of 31 genomes, including those of the six type strains of the subspecies. The results indicate the repertoire of subspecies-specific evolution among the six subspecies.

## Materials and methods

### Strains and sequences

Strains DSM 20072^T^, KCCM 34717, KCTC 3034, KCTC 3035, DSM 26046^T^, DSM 20074^T^, KCTC 13731, JCM 17838^T^, JCM 15610^T^, and DSM 20080 were obtained from the corresponding culture collections. Genomic DNA was extracted using a QIAamp DNA Mini Kit (QIAGEN, Venlo, The Netherlands). Whole-genome sequencing was performed using a PacBio RS I system (Pacific Biosciences, Menlo Park, CA, USA). The resultant raw sequencing reads were assembled using SMRT analysis v2.3.0^16^, with the HCAP.2 protocol. The constructed genome sequence was corrected using the Quiver algorithm resequencing protocol. Finally, comparative genomic analysis was performed on 31 genomes, comprising the ten genomes sequenced in this study and 21 genomes that are publicly accessible in NCBI GenBank (6 complete genomes and 15 permanent draft genomes) (Table 1). In the genome trees constructed in this study, *L. delbrueckii* subsp. *bulgaricus* PB2003/044-T3-4 and *L. delbrueckii* subsp. *delbrueckii* KCTC 13731 grouped with type strains that are different to those listed by the submitter in the NCBI database. Therefore, these strains were renamed with the subspecies they grouped into the genome tree, as follows: *L. delbrueckii* subsp. *sunkii* PB2003/044-T3-4 and *L. delbrueckii* subsp. *jakobsenii* KCTC 13731, respectively. Strain DSM 20074^T^ and KACC 13439^T^ are isogenic strains of *L. delbrueckii* subsp. *delbrueckii.* Due to the difference of genome property between the previously reported genome of KACC 13439^T^ and the newly determined genome of DSM 20074^T^, both genome sequences were included in the analyses.

**Table 1:** List of *L delbrueckii* genome sequences analyzed in this study.

Subspecies	Strain	CDSs	Size (Mb)	GC%	Level	Assembly acc no	Isolation source
*lactis*	DSM 20072^T^	2.041	2.166	49.1	Cpl	GCA_002017855.1*	Emmental cheese
	CNRZ226	1.825	1.911	50.0	Sca	GCA_000751655.1	Environment
	CNRZ327	1.901	2.105	49.8	Sca	GCA_000751695.2	Environment
	CNRZ333	1.929	2.052	49.5	Sca	GCA_000751235.1	Environment
	CNRZ700	1.940	2.086	49.5	Sca	GCA_000751275.1	Environment
	CRL581	1.891	2.137	49.6	Sca	GCA_000409675.1	Argentinian hard Cheese
	KCCM 34717	2.149	2.263	49.1	Cpl	GCA_001888905.1*	Environment
	KCTC 3034	2.122	2.238	48.9	Con	GCA_002016675.1*	Sour milk
	KCTC 3035	1.859	1.973	50.0	Cpl	GCA_001888985.1*	Unknown
	NDO2	2.009	2.132	49.6	Cpl	GCA_000182835.1	Unknown
*jakobsenii*	DSM 26046^T^	1.788	1.892	50.1	Cpl	GCA_001888925.1*	Fermented beverage
	KCTC 13731	1,812	1.911	50.1	Cpl	GCA_001888945.1*	Environment
*delbrueckii*	DSM 20074^T^	1.894	1.954	49.6	Cpl	GCA_001908495.1*	Environment
	KACC 13439^T^	1.731	1.766	50.0	Con	GCA_001263315.1	Environment
*sunkii*	JCM 17838^T^	1.833	2.004	50.1	Cpl	GCA_001888965.1*	Fermented vegetable
	PB2003/044-T3-4	1.820	1.977	50.0	Con	GCA_000179375.1	Biological product
*indicus*	JCM 15610^T^	1.956	2.022	49.4	Cpl	GCA_001908415.1*	Dairy fermented product
*bulgaricus*	ATCC 11842^T^	1.868	1.865	49.7	Cpl	GCA_000056065.1	Bulgarian yogurt
	2038	1.893	1.873	49.7	Cpl	GCA_000191165.1	Unknown
	ATCC BAA-365	1.873	1.857	49.7	Cpl	GCA_000014405.1	Unknown
	DSM 20080	1.881	1.868	49.8	Cpl	GCA_001953135.1*	Yogurt
	MN-BM-F01	1.872	1.875	49.7	Cpl	GCA_001469775.1	Traditional fermented dairy
	ND04	1.855	1.862	49.6	Cpl	GCA_002000885.1	Fermented camel milk
	CFL1	1.758	1.758	49.8	Con	GCA_001510975.1	Unknown
	CNCM I-1519	1.808	1.797	49.9	Con	GCA_000284715.1	Unknown
	CNCM I-1632	1.753	1.768	49.9	Con	GCA_000284695.1	Unknown
	Lb1-GS-1	1.755	1.743	49.9	Sca	GCA_001624925.1	Culture
	Lb1-WT	1.806	1.79	49.9	Con	GCA_001624905.1	Culture
	LBB.B5	1.764	1.778	49.8	Con	GCA_001647065.1	Home-made yogurt
	Vib27	1.875	1.853	49.8	Sca	GCA_000751635.1	Environment
	Vib44	1.844	1.818	49.7	Sca	GCA_000751895.1	Environment

A total of 31 strains were used, comprising ten strains that were sequenced in this study and 21 that were publicly accessible in NCBI GenBank (6 complete genomes and 15 permanent draft genomes).

*Cpl* Complete genome, *Sca* Scaffold, *Con* Contig. Asterisks indicate the genomes sequenced in this study.

### Gene prediction, orthologous gene clustering, and annotation

Protein coding sequences were predicted using Prodigal v.2.6.3^17^. Disrupted genes and gene fragments were identified according to the guideline of Prokaryotic Genome Annotation Guide of GenBank. Orthologous gene families were analyzed using OrthoMCL^18^, a program that utilizes all-against-all BLASTP and Markov Cluster algorithms, with an inflation value of 2.0. Pan- and core genome curves were generated using PanGP v.1.0.1^19^. The functions of gene families were annotated with BLAST search using the UniProt^20^ and COG databases^21^. The GC3 ratio was calculated using CodonW program^22^. The putative prophages in bacterial genomes were annotated and identified using PHASTER program^23^.

### Reconstruction of phylogenetic tree

For the phylogenetic analysis, the *L. amylolyticus* L6 (CP031835) and *L. acetotolerans* NBRC 13120 (AP014808) genomes were used as the two outgroups. Of the orthologous genes found in the core genome, those with a single copy in each genome were selected and used to infer the phylogenomic tree. MUSCLE v3.8.31^24^ was used to align the amino acid sequences of the genes. Aligned positions that showed gaps in > 50% of the strains across all 33 genomes (including the outgroups) were removed using Gblocks v0.91^25^. The final gene alignments were concatenated using FASconCAT^26^ to generate concatamers. To select an appropriate evolution model, a model test was performed using ProtTest v3.2^27^. A maximum-likelihood tree was constructed using RAxML v8.2.4^28^. All phylogenetic trees were viewed using Dendroscope v3.2.2^29^. To estimate the genome sequence similarities, the average nucleotide identity (ANI) was calculated using OrthoANI^30^. The resultant ANI distance was ordinated using the heatmap plot function of the R program.

### Analysis of the gene gain and loss of gene families

To calculate gain and loss events and turnover rates in gene families, the BadiRate software^31^ was used. The orthologous gene tables and maximum-likelihood tree obtained in the above phylogenetic analyses were used. To evaluate the proper evolutionary model, two different branch models (global-rates and free-rates models) and three kinds of stochastic population models (Gain-and-Death, Birth–Death-Innovation, and Lambda-Innovation models) were evaluated. The goodness of fitness of these models was assessed by likelihood values. To analyze the degree of horizontal gene transfer (HGT) in each subspecies, a binary matrix of the presence or absence of ortholog genes was computed, and a network tree was generated using SplitTree v4.14.5^32^.

## Results and discussion

### Genomic characteristics of *L. delbrueckii*

Analysis of 31 *L. delbrueckii* genomes determined that this group has a genome size of 1.93 ± 0.16 Mb and a G + C content of 49.8 ± 0.4% (Table 1). The average genome size and G + C content across the *Lactobacillus* genus listed on GenBank were 1.96 Mb and 37.2%, respectively. This indicates that the *L. delbrueckii* group has genomes that are near-average in size in the genus. When the genome sizes were compared among subspecies lineages, the *lactis* lineage was found to have the largest genome size (2.1 Mb), and the *bulgaricus* lineage was found to have the smallest genome size (1.85 Mb) (Fig. S1). Genome reduction in the *bulgaricus* subspecies compared to *lactic* subspecies reported in previous study^7^ was also verified in this study. Although *L. delbrueckii*, particularly the *lactis* lineage, showed an overall increase in gene count, it is difficult to say that this corresponded to an expansion of its genetic content because this increase was driven by mobile elements and gene fragmentation.

The core genome comprised 1,069 orthologous gene families, and the pan-genome was an open pan-genome that consisted of 4,332 orthologous gene families (Fig. S2). According to the prediction of PanGP program, the subsequent addition of a new genome sequence to this species would be expected to result in the addition of 30–45 new gene families to the pan-genome.

Although it is known that the genome G + C content is typically directly proportional to the genome size in prokaryotes^33^, the results obtained from *L. delbrueckii* in this study diverged from this trend. In a previous report, Guchte et al.^8^ showed that the exceptionally high GC content in *L. delbrueckii* supsp. *bulgaricus* ATCC 11842^T^ is mainly due to the difference at codon position 3 (GC3), and based on that they suggested that this subspecies is in active phase of evolution. Their finding is also confirmed in this study. While the G + C content was evenly in the range of 49.4–50.1% across the subspecies, the GC3 values were high as 57.0–61.1% in all subspecies (Table S1). Thus the GC3 difference can be said a characteristic of *L. delbrueckii* strains. O′Sullivan et al. suggested that the reason of the high G + C content in *L. delbrueckii* is a recent lateral gene transfer event between two distantly related species occupying the same environmental niche^34^. Considering together, the active evolution through lateral gene transfer may be the reason of the high level of G + C *L. delbrueckii* subspecies.

### Phylogeny of *L. delbrueckii* subspecies

**Figure 1:**
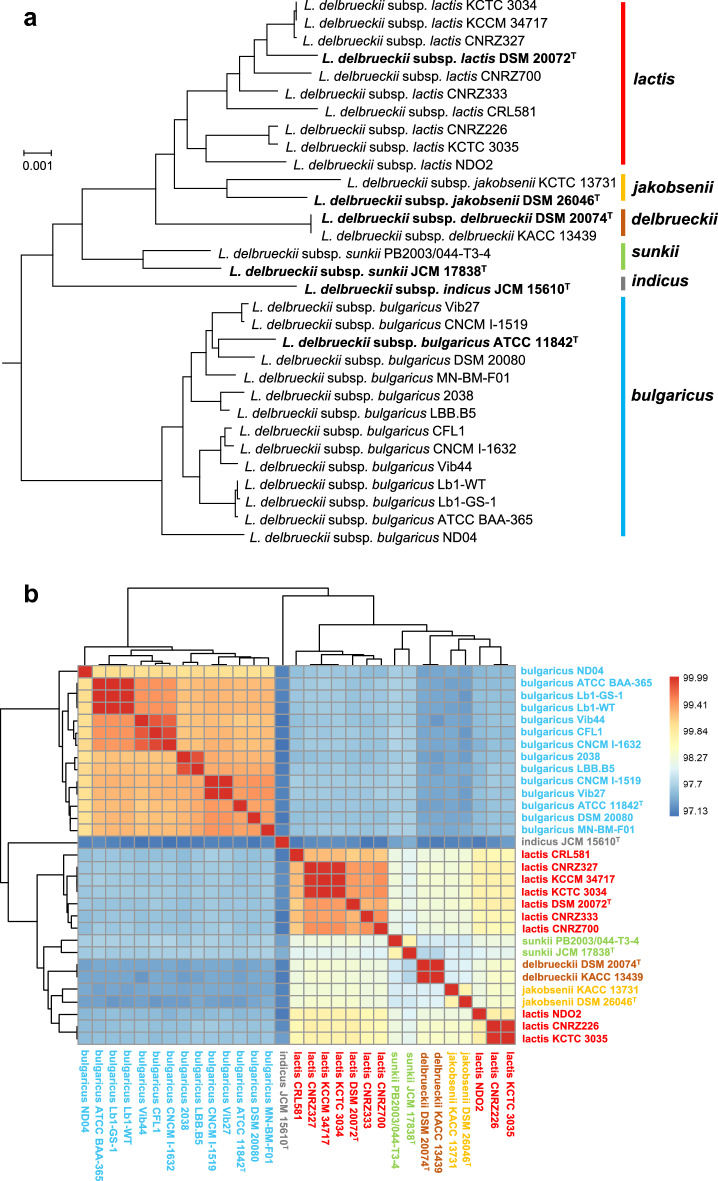
Genomic distance and relationship among *L. delbrueckii* subspecies*.* (**a**) Maximum-likelihood tree constructed using amino acid sequences of 689 core genes. The tree was rooted using the outgroups *Lactobacillus amylolyticus* L6 and *Lactobacillus acetotolerans* NBRC 13,120. The scale represents the number of substitutions per site. The bootstrap values were 100% in all nodes. (**b**) Average nucleotide identity (ANI), demonstrating the genomic distance among *L. delbrueckii* subspecies. The ANI distance was plotted as a heatmap.

**Figure 2:**
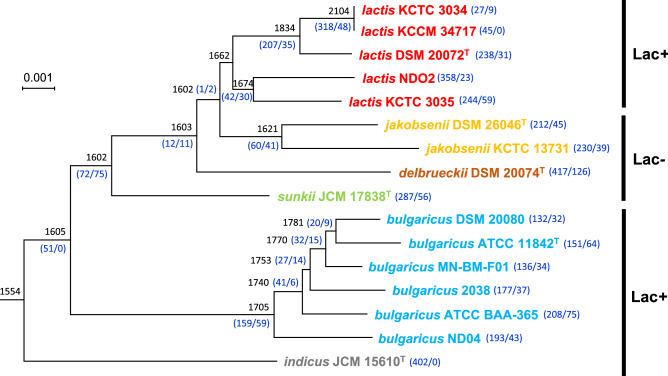
Analysis of ancestral genes and minimal gene gain/loss among the complete genome sequences. Black numbers adjacent to the internal nodes indicate the number of estimated ancestral genes. Blue numbers on the branches denote the minimum number of gains and losses under the best fit model. ‘Lac + ’ and ‘Lac−’ indicate the presence and absence of lactose fermentation capability.

A total of 689 single-copy core genes were extracted from the 31 *L. delbrueckii* genomes included in the study, the amino acid sequences of which were then aligned and concatenated to create an aligned sequence that spanned 215,261 aa positions from each genome. A maximum-likelihood tree was constructed from the obtained sequences using the WAG-I-G-F model. The results of this analysis indicated the independent evolutionary lineages depending on the six subspecies (Fig. 1a). Unlike the other subspecies, the subspecies *bulgaricus* was the only lineage that was found to contain homogeneous strains that had diverged forming a deep branch close to the universal common ancestor of this species and had evolved independently. In contrast, the other five subspecies are highly diverse, heterogeneous lineages. The *lactis*-*jakobsenii*-*delbrueckii*-*sunkii* (LJDS) subspecies formed a loose clade in all trees analyzed in this study (Fig. 1a), but the genome sequence distance calculated from the ANI values among the four subspecies were large enough to support the independent subspecies status (Fig. 1b). The *indicus* lineage was found to have unstable branching positions depending on the tree building option. Depending on the gene set and genome set chosen for tree reconstruction, the *indicus* lineage branched as a sister group of the LJDS clade as appeared in the Fig. 1 or separated from the other five subspecies forming a distinct deepest branch as appeared in Fig. 2.

### Gene gain and loss events

After the likelihood scores were calculated using a model test (Table S2), the BDI-FR-ML model was chosen to calculate the gene turnover rates and gene gain and loss events. The results of this analysis indicated that the most recent common ancestor of *L. delbrueckii* had 1,554 genes and that the number of genes increased throughout the evolutionary history of the species. In particular, the gene count increased markedly in the branch of the *lactis* lineage that included KCTC 3034, KCCM 34717, and DSM 20072^T^ as presented in the complete genome tree (Fig. 2). However, because the increase in the number of genes involved gene fragmentation or the multiplication of transposons and analog of mobile elements, an increasing gene count does not necessarily imply genome expansion. Regarding outlier events, which had significantly higher gain and loss rates than would be expected based on the corresponding branches, 47 outlier events were found in 19 of the 30 branches, including internal and external branches. Of the 47 outlier events, 41 were gain events, of which 34 were found in the LJDS lineage. The remaining seven events were found in the *bulgaricus* lineage. Most (> 70%) of the outlier events corresponded to transposons, gene fragmentation, and hypothetical proteins. The remaining events (14 events; 29.8%) were found to be due to the turnover of fragmented and hypothetical proteins. Collectively, these results suggested that the multiplication and loss of transposons occurred frequently. In a similar manner, the number of prophage gene sequences found in LJDS lineage was 3.3 on average while it was 1.8 in bulgaricus lineage (Table S1). In detail, the lactis subspecies showed the highest value (3.8 prophage genes predicted per genome on average) as twice many as the bulgaricus subspecies. This suggests that the increase in the gene count of *L. delbrueckii* was driven by mobile elements and gene fragmentation, and that such increases were most frequent in the *lactis* lineage. These results are similar to the previous report of Kafsi et al. 2014^7^.

### HGT analysis

**Figure 3:**
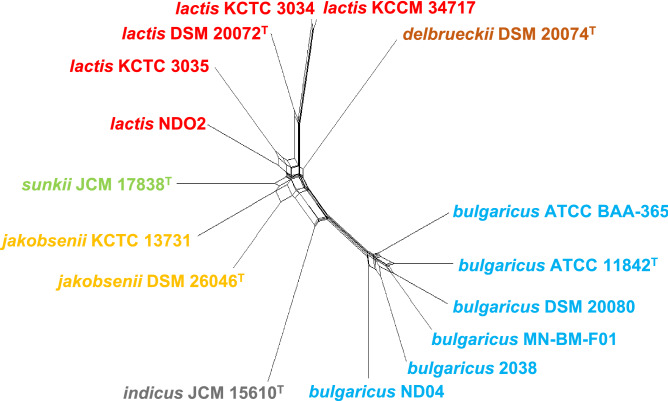
Network tree showing the HGT events among *L. delbrueckii* subspecies. The network tree of complete genomes was generated using the neighbor-net algorithm based on a binary matrix of the presence or absence of gene families generated by the OrthoMCL program. Splits in the tree show the possibility of non-vertical evolution between branches.

To investigate possible occurrences of HGTs between different lineages, a split decomposition analysis was performed using complete genome sequences based on the presence of ortholog genes. The resultant network tree indicated that the *bulgaricus* lineage had frequent gene transfers, but these only occurred within the subspecies (Fig. 3). Frequent gene transfer occurred when the lineage first diverged from the members of the LJDS lineage. Gene transfer between the LJDS and *bulgaricus* lineages did not occur frequently. This implies that the *bulgaricus* lineage evolved independently of the other subspecies.

### Carbon sources used for fermentation

The carbon source catabolism was predicted using KEGG Pathway tool (Table S3). The predicted sugar fermenting pathway was generally in good agreement with previous experimental data reported in the original description of the four subspecies type strains^10–12^. Based on the genome analyses, the capability for sugar metabolism differs between the subspecies. The capability for lactose fermentation was preserved in the genomes of the *bulgaricus* lineage, as has been reported in original description^10^ and previous genome report^7^. Kafsi et al. stated that the lactose fermenting capacity of subspecies *bulgaricus* relies on horizontally acquired rather than deep ancestral genes^7^. In our genome analyses, lactose fermentation capability was absent in the common ancestor of the LJDS lineage but reappeared in the *lactis* lineage (Fig. 2). This suggests that the capability for lactose fermentation in *L. delbrueckii* subsp. *lactis* either arose via HGT as reported in the subspecies *bulgaricus* or that the remaining members of the LJDS lineage lost genes that were present in the common ancestor of both the LJDS and *bulgaricus* lineages. Members of the LJDS lineage can be distinguished from the *bulgaricus* lineage via the high number of sugars they use rather than by the carbon type used (Table S3), in accordance with the characteristics of the subspecies lactis reported previously. This may be due to the high intra-subspecies genetic diversity found within the LJDS lineage. Genes for the metabolism of lactose and other sugars may have led to the ecological niche specialization of the subspecies. Actually, the ratio of dairy and environmental strains was low as 38% in LJDS lineage (5 dairy and 8 environmental strains), while the isolation source of subspecies *bulgaricus* was mainly restricted to dairy products (71%; 5 dairy and 2 environmental strains) (Table 1).

### Amino acid metabolism

In the earlier genome report of Kafsi et al., the amino acid biosynthesis capacities are more severely reduced in the ssp. *bulgaricus* than in the ssp. *lactis*^7^. According to Kafsi et al., the *bulgaricus* lineage have evolved the ability to strengthen their transport systems for the uptake of peptides and oligopeptides from the outside environment rather than by the synthesis of peptides and have adapted to peptide- and vitamin-rich environments such as fermented milk, and to have evolved to survive solely from lactose utilization and amino acid salvage from a small subset of sugars^7^. We also observed that some amino acid synthesis pathways (arginine and proline) were inactivated from the *bulgaricus* lineage but preserved in *lactis* lineage (Table S3). Instead, methionine is preserved in the subspecies *bulgaricus*, *jakobsenii*, and *sunkii* only.

### Subspecies-specific characteristics

#### Subspecies bulgaricus

As shown in Table S4, the *bulgaricus* lineage gained the homocysteine S-methyltransferase gene (OG11897) required for methionine salvage from homocysteine at the time of its divergence into *L. delbrueckii* subsp. *bulgaricus*. The sequence of this gene was more similar to that of *L. helveticus* (99%) than the homocysteine S-methyltransferase found in *L. delbrueckii* and was found to have been truncated. The transport of other proteins and amino acids by multiple ABC-type peptide and oligopeptide transport systems (OG11756, OG11762, OG11783, OG11784, OG11956, OG11986) increased in this lineage. Genes for pyruvate and water dikinases (ppsA, OG OG10047, OG11691, OG11781) that convert pyruvate to phosphoenolpyruvates were also observed to increase in number. Their alignments with the original genes suggested that some pyruvate utilizing enzymes (OG11691, OG11781) were fragmented in the *bulgaricus* lineage, resulting in an increase in the number of genes. While the OG10047 was the basic and preserved enzyme in LJDS and indicus, it was fragment in OG10047. Instead of the fragmented OG10047, some bulgaricus strains possessed alternative ppsA (OG12091, OG13520). Therefore, it appears that the *bulgaricus* lineage has lost its ability to convert pyruvate to phosphoenolpyruvate. Furthermore, genes related to dNTP-sugar synthesis and the Leloir pathway for galactose metabolism (d-TDP-4-dehydrorhamnose reductase, OG11366; dTDP-4-dehydro-rhamnose 3,5-epimerase, OG11367; galactose-1-phosphate uridylyltransferase, OG11658; and galactokinase, OG11659), as well as those contributing to the arginine deiminase pathway (OG11487), appear to have been lost in this lineage. Genes in the arginine deiminase pathway were retained in the other five subspecies.

#### Subspecies lactis

Various glycosidases, including orthologs of alpha-glucosidase, and sugar transport PTS system have been inserted into the genome over the course of evolution since the divergence of the LJDS lineage. The genes that that contribute to glycerol degradation and lactose degradation appear to have been inserted into the genome since the subspecies *lactis* diverged (Table S5). Genes related to lactose-specific (OG11661) and mannitol-specific (OG11504) phosphotransferase systems (PTS) systems that were lost when the LJDS lineage diverged appear to have been re-inserted into the genome when the *lactis* lineage diverged.

#### Subspecies jakobsenii

In the *jakobsenii* lineage, genes that encode proteins that contribute to branched-chain amino acid transport (OG12143-OG12144 and OG12325-OG12326), were inserted into the genomes (Table S6). In contrast, genes that encode organic compound transport proteins, such as lactose permease (OG11353), and those that contribute to vitamin (riboflavin) synthesis (OG11270 and OG11609) appear to have been lost.

#### Subspecies delbrueckii

Genes that contribute to nitrate and nitrite metabolism and nitrogen fixation were not found in any of the completely sequenced *L. delbrueckii* genomes. Most of subspecies of *L. delbrueckii* secure nitrogen sources via amino acid and peptide salvage pathways. The *delbrueckii* subspecies was found to contain strain-specific glucansucrases (OG12198), in addition to the inulosucrase (OG11707) and glucansucrase (OG12198) already present in the LJDS lineage (Table S7). However, it appears that genes that contribute to the metabolism of mannitol (OG11132 and OG11294), trehalose (OG11545), and galactose (OG11658) were lost.

#### Subspecies sunkii

With divergence into the JCM 17838^T^ strain, genes encoding dihydroxyacetone kinase (OG11728-OG11730), alpha-galactosidase (OG11950), and glycogen synthase (OG13271), which are enzymes that contribute to glycerol and sugar metabolism, appear to have been inserted into the genome (Table S8). However, the lineage lost the gene families that are responsible for the transport of amino acids (OG11438-OG11440), peptides and oligopeptides (OG11638 and OG11717).

#### Subspecies indicus

With divergence into the JCM 15610^T^ strain, this lineage has gained various genes that are related to ABC-type transport systems (OG10619 and many other genes) and sugar or sugar-alcohol PTS (OG11615, OG11661, OG11721, and OG11828) (Table S9). Furthermore, the LarABCDE gene operon (OG13096-OG13101) that is responsible for lactate racemization, converting d-form lactate into its l-form, was observed in this lineage but not in other *L. delbrueckii* lineages, suggesting that it was introduced to the genome via HGT. The lineage also gained the genes for proteins that contribute to lactose and galactose degradation (from galactose-6-phosphate to glyceraldehyde 3-phosphate) (OG11716 and OG11820).

## Conclusions

The species *L. delbrueckii* has gained genetic diversity via horizontal gene transfer between subspecies, and has increased its genome size. Such adaptability has made it an economically important species with extensive industrial application. The subspecies *bulgaricus* is a homogeneous group that diverged from other subspecies a long time ago and has subsequently evolved independently. The relatively small genome size of the *bulgaricus* compared to other subspecies suggests that it has experienced genome reduction during its evolutionary history and is currently becoming specialized in lactose fermentation. Active HGT and an evolutionary trend for increasing genome size have been observed in the subspecies *lactis*, *jakobsenii*, *delbrueckii*, and *sunkii*. These phenomena appear to be a way of gaining genetic diversity to adapt to various novel natural environments and carbon sources. The long-term adaptation of specialized strains to their environments may have led to genome reduction and intraspecific diversification through various mechanisms. Thus, subspeciation in *L. delbrueckii* may have been driven by the availability of carbon sources. The *indicus* lineage seems to be evolving independently of the other five subspecies. Some subspecies currently only have one known strain, and this limited the potential for this study to understand the characteristics of all six subspecies of *L. delbrueckii*. It is anticipated that as more strains of subspecies *indicus*, *sunkii*, *delbrueckii*, and *jakobsenii* are discovered, it will become easier to understand the characteristics and evolutionary processes of each subspecies more systematically.

## Supplementary Information

Supplementary Information 1.

Supplementary Information 2.

Supplementary Information 3.

## Data Availability

The genome sequences generated during the current study are available from the NCBI under the accession numbers GCA_001888905.1, GCA_001888925.1, GCA_001888945.1, GCA_001888965.1, GCA_001888985.1, GCA_001908415.1, GCA_001908495.1, GCA_001953135.1., GCA_002016675.1, and GCA_002017855.1.
