# Supplementary material for: Subspecies-level genome comparison of Lactobacillus delbrueckii
Source: Sci Rep. 2023 Feb 23;13:3171. doi: 10.1038/s41598-023-29404-3 (PMC9950072; doi:10.1038/s41598-023-29404-3)
Supplement: Supplementary file 1 — Supplementary Information 1. [file 41598_2023_29404_MOESM1_ESM.pptx]

## Slide 1
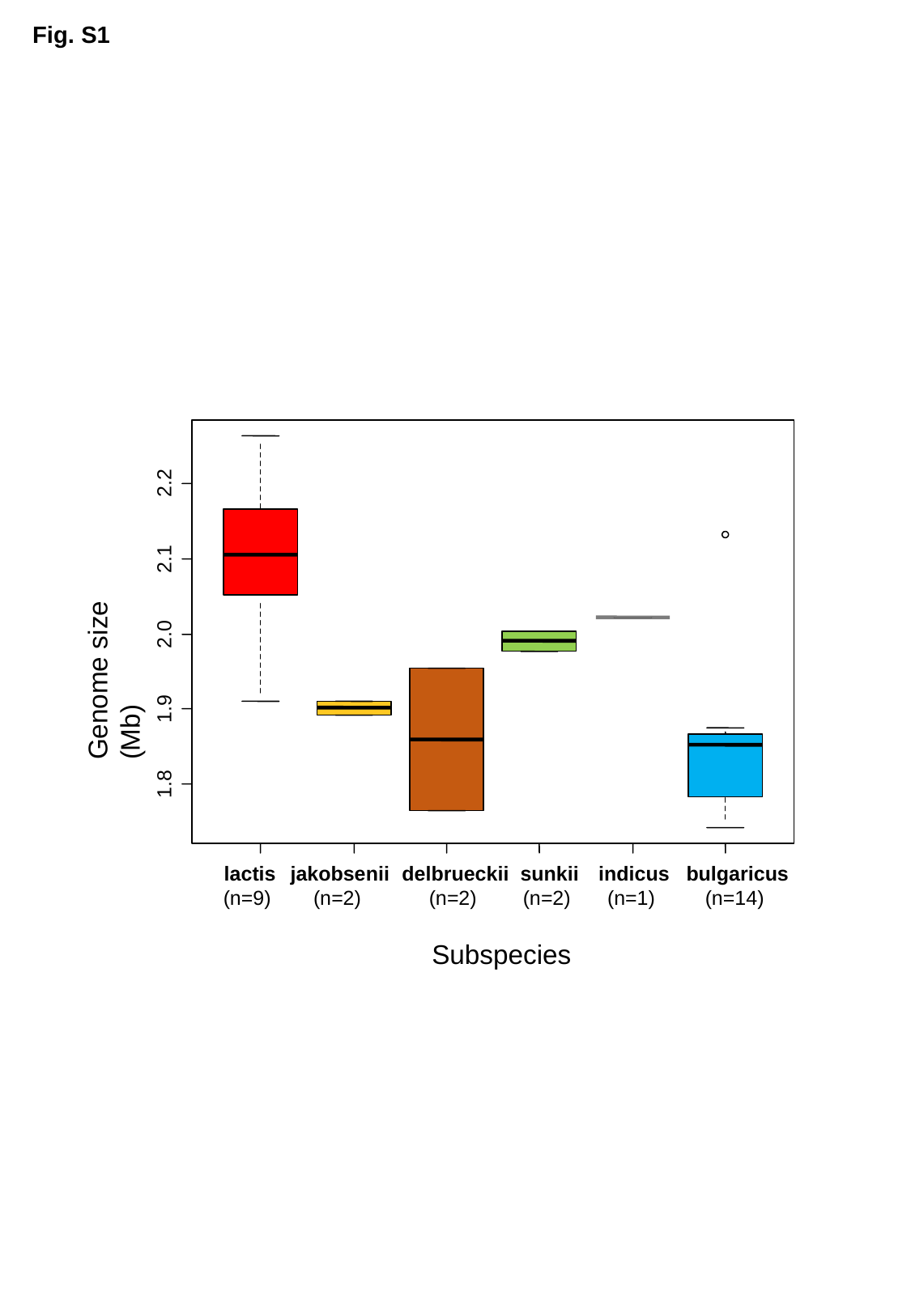

Fig. S1
2.2
2.1
2.0
Genome size (Mb)
1.9
1.8
lactis
(n=9)
jakobsenii
(n=2)
delbrueckii
(n=2)
sunkii
(n=2)
indicus
(n=1)
bulgaricus
(n=14)
Subspecies
