# Supplementary material for: Subspecies-level genome comparison of Lactobacillus delbrueckii
Source: Sci Rep. 2023 Feb 23;13:3171. doi: 10.1038/s41598-023-29404-3 (PMC9950072; doi:10.1038/s41598-023-29404-3)
Supplement: Supplementary file 2 — Supplementary Information 2. [file 41598_2023_29404_MOESM2_ESM.pptx]

## Slide 1
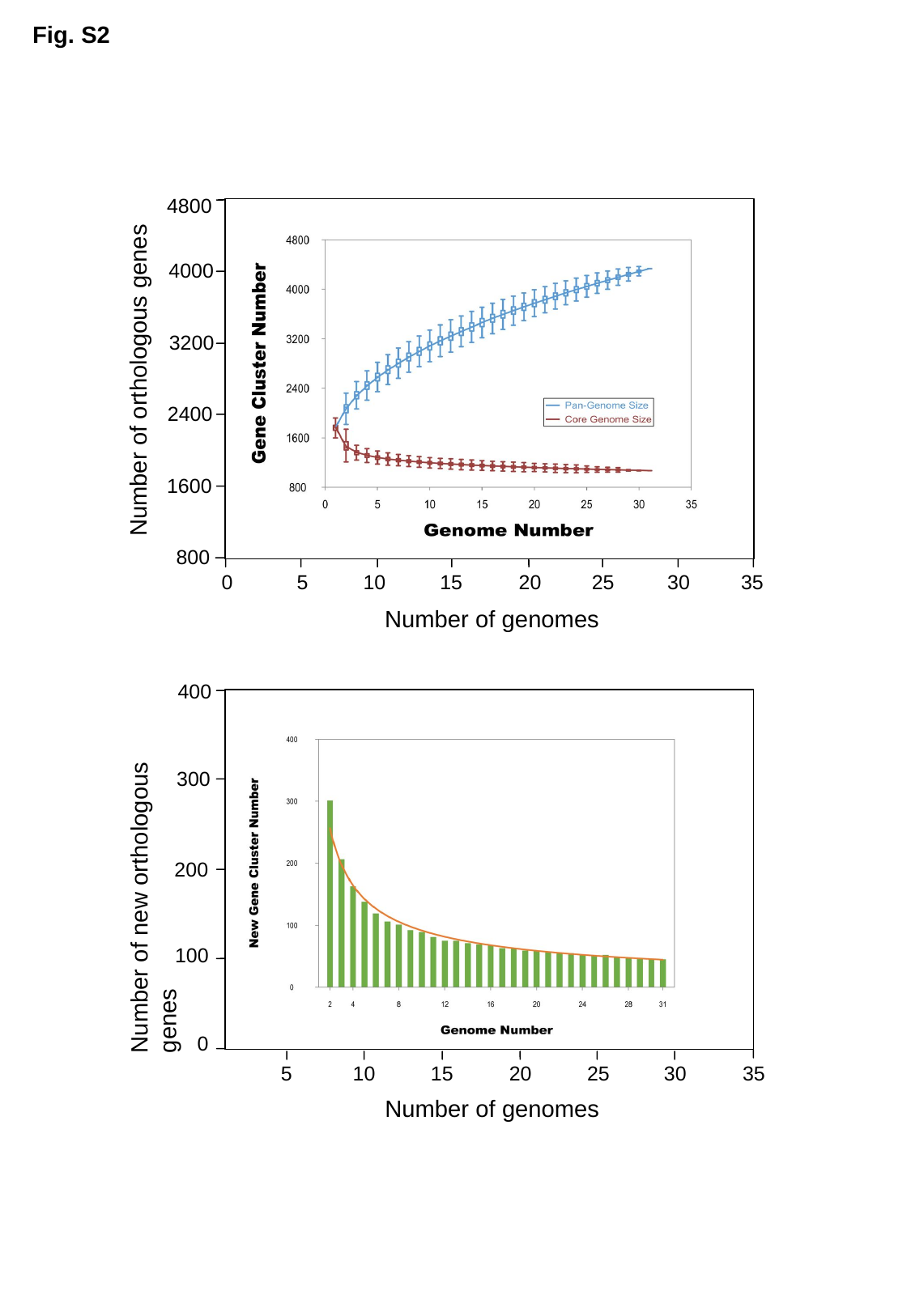

Fig. S2
4800
4000
3200
Number of orthologous genes
2400
1600
800
0
5
10
15
20
25
30
35
Number of genomes
400
300
200
Number of new orthologous genes
100
0
5
10
15
20
25
30
35
Number of genomes
