# Supplementary material for: Subspecies-level genome comparison of Lactobacillus delbrueckii
Source: Sci Rep. 2023 Feb 23;13:3171. doi: 10.1038/s41598-023-29404-3 (PMC9950072; doi:10.1038/s41598-023-29404-3)

# Subspecies-level genome comparison of *Lactobacillus delbrueckii*

Min-gyung Baek^1,2^, Kwan Woo Kim^1,2^, Hana Yi^1,3*^

^1^ Interdisciplinary Program in Precision Public Health, Korea University, Seoul, Korea

^2^ Department of Public Health Sciences, Korea University, Seoul, Korea

^3^ School of Biosystems and Biomedical Sciences, Korea University, Seoul, Korea

*Corresponding author: Professor Hana Yi

E-mail: [hanayi@korea.ac.kr](mailto:hanayi@korea.ac.kr)

**Supplementary table 1. GC ratio and frequency of predicted intact, incomplete, and questionable prophage gene sequences in the bacterial genomes.**

| **Subspecies** | **Strain** | **GC content (%)** | | **Prophage gene sequences** | | | | |
| --- | --- | --- | --- | --- | --- | --- | --- | --- |
|  |  | **GC** | **GC3** | | **Intact** | **Incomplete** | **Questionable** | **Total** |
| *lactis* | DSM 20072^T^ | 49.1 | 60.2 | | 1 | 3 | 0 | 4 |
|  | CNRZ226 | 50.0 | 60.7 | | 0 | 1 | 1 | 2 |
|  | CNRZ327 | 49.8 | 59.8 | | 0 | 3 | 0 | 3 |
|  | CNRZ333 | 49.5 | 60.4 | | 0 | 3 | 0 | 3 |
|  | CNRZ700 | 49.5 | 59.6 | | 0 | 2 | 0 | 2 |
|  | CRL581 | 49.6 | 60.6 | | 0 | 2 | 0 | 2 |
|  | KCCM 34717 | 49.1 | 59.2 | | 0 | 5 | 3 | 8 |
|  | KCTC3034 | 48.9 | 58.8 | | 0 | 5 | 2 | 7 |
|  | KCTC3035 | 50.0 | 60.9 | | 0 | 0 | 1 | 1 |
|  | NDO2 | 49.6 | 60.4 | | 1 | 3 | 2 | 6 |
| *jakobsenii* | DSM 26046^T^ | 50.1 | 52.8 | | 0 | 2 | 0 | 2 |
|  | KCTC 13731 | 50.1 | 61.2 | | 0 | 4 | 1 | 5 |
| *delbrueckii* | DSM 20074^T^ | 49.6 | 60.4 | | 0 | 4 | 0 | 4 |
|  | KACC 13439^T^ | 50.0 | 61.8 | | 0 | 1 | 0 | 1 |
| *sunkii* | JCM 17838^T^ | 50.1 | 61.4 | | 0 | 1 | 0 | 1 |
|  | PB2003/04-T3-4 | 50.0 | 59.8 | | 0 | 2 | 0 | 2 |
| *indicus* | JCM 15610^T^ | 49.4 | 59.6 | | 0 | 2 | 1 | 3 |
| *bulgaricus* | ATCC 11842^T^ | 49.7 | 60.9 | | 0 | 3 | 0 | 3 |
|  | 2038 | 49.7 | 60.8 | | 0 | 3 | 0 | 3 |
|  | ATCC BAA-365 | 49.7 | 61.0 | | 0 | 2 | 0 | 2 |
|  | DSM 20080 | 49.8 | 60.4 | | 0 | 1 | 0 | 1 |
|  | MN-BM-F01 | 49.7 | 60.4 | | 0 | 2 | 0 | 2 |
|  | ND04 | 49.6 | 59.9 | | 0 | 1 | 0 | 1 |
|  | CFL1 | 49.8 | 60.2 | | 0 | 2 | 0 | 2 |
|  | CNCM I-1519 | 49.9 | 60.0 | | 1 | 1 | 0 | 2 |
|  | CNCM I-1632 | 49.9 | 60.5 | | 0 | 1 | 0 | 1 |
|  | Lb1-GS-1 | 49.9 | 60.6 | | 0 | 1 | 0 | 1 |
|  | Lb1-WT | 49.9 | 61.2 | | 0 | 1 | 0 | 1 |
|  | LBB.B5 | 49.8 | 60.7 | | 0 | 1 | 0 | 1 |
|  | Vib27 | 49.8 | 60.7 | | 1 | 1 | 0 | 2 |
|  | Vib44 | 49.7 | 60.4 | | 1 | 1 | 1 | 3 |

**Supplementary table 2. Likelihood values according to the branch models.**

GD, Gain-and-Death stochastic model; BDI, Birth-Death-and-Innovation stochastic model; LI, Lambda-Innovation model; GR, global-rates model; FR, free-rates model; ML, maximum-likelihood.

| **Branch model** | **Log-Likelihood score** |
| --- | --- |
| GD-GR-ML | -25,505.60 |
| LI_GR_ML | -24,940.36 |
| BDI_GR_ML | -24,853.09 |
| GD-FR-ML | -25,106.17 |
| LI_FR_ML | -24,356.28 |
| BDI_FR_ML | -24,065.54 |

**Supplementary table 3. Sugar fermenting and amino acid synthesis capacities inferred from KEGG Pathway.**

Presence of complete pathway is indicated as + and the absence or incomplete pathway is indicated as -.

| **Subspecies** | ***bulgaricus*** |  |  |  |  |  | ***lactis*** |  |  |  |  | ***jakobsenii*** |  | ***delbrueckii*** | ***sunkii*** | ***indicus*** |
| --- | --- | --- | --- | --- | --- | --- | --- | --- | --- | --- | --- | --- | --- | --- | --- | --- |
| Strain | ATCC 11842 | ATCC BAA-365 | DSM 20080 | 2038 | ND04 | MN-BM-F01 | KCTC 3034 | KCTC 3035 | KCCM 34717 | DSM 20072 | ND02 | DSM 26046 | KCTC 13731 | DSM 20074 | JCM 17838 | JCM 15610 |
| **Sugar fermentation** | | | | | | | | | | | | | | | | |
| D-Glucose | + | + | + | + | + | + | + | + | + | + | + | + | + | + | + | + |
| Arbutin | + | - | + | + | - | - | + | + | + | + | + | + | + | + | + | + |
| Salicin | + | - | + | + | - | - | + | + | + | + | + | + | + | + | + | + |
| D-Fructose | + | + | + | - | - | + | + | + | + | + | + | + | + | + | + | + |
| D-Mannose | + | + | + | + | + | + | + | + | + | + | + | + | + | + | + | + |
| D-Galactose | - | + | + | + | + | + | + | + | + | + | + | - | + | - | - | + |
| Lactose | + | + | + | + | + | + | + | + | + | + | + | - | - | - | - | + |
| D-Raffinose | - | - | - | - | - | - | - | - | - | - | + | - | - | - | + | - |
| Sucrose | + | - | + | + | + | + | + | + | + | + | + | + | + | + | + | + |
| Maltose | - | - | - | - | - | - | - | - | - | + | + | + | + | + | + | - |
| Trehalose | - | - | - | - | - | - | + | + | + | + | + | + | + | + | + | - |
| Cellobiose | + | + | + | + | + | + | + | + | + | + | + | + | + | + | + | + |
| **Amino acid synthesis** | | | | | | | | | | | | | | | | |
| Threonine | + | + | + | + | + | + | + | + | + | + | + | + | + | - | + | + |
| Cysteine | + | + | + | + | + | + | + | + | + | + | + | + | + | - | - | + |
| Lysine | - | - | + | + | + | + | + | + | + | + | + | + | + | - | + | + |
| Arginine | - | - | - | - | - | - | + | + | + | - | + | + | + | - | + | + |
| Proline | - | - | - | - | - | + | + | + | + | - | + | + | + | + | + | - |
| Methionine | - | - | + | + | + | + | - | - | - | - | - | + | + | - | + | - |

**Supplementary table 4. Orthologous genes gained/lost in the *bulgaricus* lineage.**

| 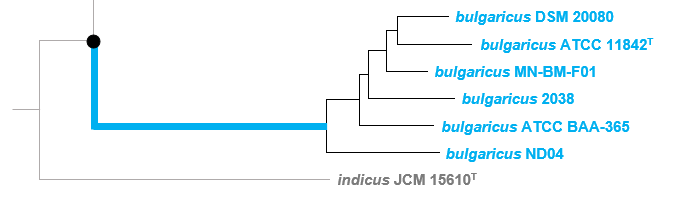 | | | | | | |
| --- | --- | --- | --- | --- | --- | --- |
| **Evolution path** | | **Gain/**  **loss** | **Orthologous gene_Fam** | **Annotation** | **COG No.** | **COG cat.** |
| **From** | **To** |  |  |  |  |  |
| Common ancestor of *lactis-jakobsenii-delbrueckii-sunkii* and *bulgaricus* lineages | *bulgaricus* lineage | Gain | OG10024 | Carbamoyl-phosphate synthase, large chain | COG0458 | EF |
|  |  |  | OG10031 | Endopeptidase | COG3590 | O |
|  |  |  | OG10034 | Possible esterase |  |  |
|  |  |  | OG10036 | Predicted membrane protein | COG3274 | S |
|  |  |  | OG10040 | ABC-type antimicrobial peptide transport system, ATPase component | COG1136 | V |
|  |  |  | OG10044 | Putative phosphotyrosine protein phosphatase | COG0394 | T |
|  |  |  | OG10045 | ABC-type antimicrobial peptide transport system, permease component | COG0577 | V |
|  |  |  | OG10046 | Ribosomal-protein-serine acetyltransferase, putative | COG1670 | J |
|  |  |  | OG10047 | Pyruvate, water dikinase | COG0574 | G |
|  |  |  | OG10052 | Alcohol-acetaldehyde dehydrogenase | COG1454 | C |
|  |  |  | OG10054 | Methyltransferase | COG0500 | QR |
|  |  |  | OG10055 | Putative multidrug efflux protein | COG0534 | V |
|  |  |  | OG10057 | Transport permease protein | COG0842 | V |
|  |  |  | OG10061 | Lysine-specific permease | COG0833 | E |
|  |  |  | OG10066 | Putative fumarate reductase (Flavoprotein) | COG1053 | C |
|  |  |  | OG10074 | Homoserine O-succinyltransferase | COG1897 | E |
|  |  |  | OG10082 | ABC transporter, ATP-binding/permease protein | COG1132 | V |
|  |  |  | OG10102 | Carbamoyl-phosphate synthase |  |  |
|  |  |  | OG10122 | Peptide binding protein | COG4166 | E |
|  |  |  | OG10614 | Diguanylate cyclase (GGDEF) domain protein | COG2199 | T |
|  |  |  | OG11337 | Serine/threonine protein kinase | COG0515 | RTKL |
|  |  |  | OG11347 | ABC transporter, ATP-binding protein | COG1132 | V |
|  |  |  | OG11391 | Permease of the major facilitator superfamily | COG0477 | GEPR |
|  |  |  | OG11405 | ABC transporter, ATP-binding and permease protein | COG1132 | V |
|  |  |  | OG11414 | Permease | COG2814 | G |
|  |  |  | OG11441 | LysR substrate binding domain |  |  |
|  |  |  | OG11461 | Oxidoreductase | COG0702 | MG |
|  |  |  | OG11463 | DNA invertase | COG2452 | L |
|  |  |  | OG11508 | ABC-type multidrug transport system, ATPase component | COG4586 | R |
|  |  |  | OG11511 | Antitoxin HicB |  |  |
|  |  |  | OG11516 | Peptide binding protein | COG4166 | E |
|  |  |  | OG11517 | Sensory box/GGDEF family protein | COG2200 | T |
|  |  |  | OG11522 | Serine acetyltransferase | COG1045 | E |
|  |  |  | OG11525 | Signal transduction diguanylate cyclase | COG2199 | T |
|  |  |  | OG11530 | LPS biosynthesis protein | COG3475 | M |
|  |  |  | OG11540 | Fumarate reductase, flavoprotein subunit, N-terminally truncated | COG3976 | S |
|  |  |  | OG11569 | Toxin HicA |  |  |
|  |  |  | OG11576 | Alpha/beta hydrolase superfamily protein | COG4814 | R |
|  |  |  | OG11578 | Integrase-like protein | COG0582 | L |
|  |  |  | OG11584 | EpsIIL, Putative polysaccharide repeat unit transport protein | COG2244 | R |
|  |  |  | OG11614 | Glycosyltransferase | COG1442 | M |
|  |  |  | OG11618 | Signal transduction diguanylate cyclase |  |  |
|  |  |  | OG11620 | Peptide binding protein | COG4166 | E |
|  |  |  | OG11642 | Glycosyltransferase related enzyme | COG0463 | M |
|  |  |  | OG11650 | CRISPR-associated endonuclease Cas1 | COG1518 | L |
|  |  |  | OG11671 | ABC-type uncharacterized transport system, permease component |  |  |
|  |  |  | OG11672 | ABC transporter, permease protein | COG3694 | R |
|  |  |  | OG11674 | Transposase |  |  |
|  |  |  | OG11675 | Pyridoxine 5'-phosphate oxidase V related favin-nucleotide-binding protein | COG3576 | R |
|  |  |  | OG11681 | Putative ABC transporter, ATP-binding protein | COG1116 | P |
|  |  |  | OG11686 | Ribonuclease H1 | COG0328 | L |
|  |  |  | OG11691 | Pyruvate, water dikinase (fragment) | COG0574 | G |
|  |  |  | OG11710 | CRISPR-associated endoribonuclease Cas2 | COG3512S | S |
|  |  |  | OG11711 | CRISPR-associated protein |  |  |
|  |  |  | OG11742 | Transposase | COG2826 | L |
|  |  |  | OG11744 | Amino acid permease | COG0531 | E |
|  |  |  | OG11745 | Oligopeptide binding protein | COG4166 | E |
|  |  |  | OG11746 | Oligopeptide binding protein | COG4166 | E |
|  |  |  | OG11747 | Chorismate mutase | COG1605 | E |
|  |  |  | OG11756 | Peptide ABC transporter substrate-binding protein | COG4166 | E |
|  |  |  | OG11762 | Peptide ABC transporter substrate-binding protein | COG4166 | E |
|  |  |  | OG11774 | Predicted membrane protein |  |  |
|  |  |  | OG11776 | Oligopeptide binding protein | COG4166 | E |
|  |  |  | OG11781 | Pyruvate, water dikinase (fragment) | COG0574 | G |
|  |  |  | OG11783 | Peptide ABC transporter substrate-binding protein | COG4166 | E |
|  |  |  | OG11784 | Peptide ABC transporter substrate-binding protein | COG4166 | E |
|  |  |  | OG11788 | Amino acid permease | COG1113 | E |
|  |  |  | OG11791 | Alpha-amylase | COG0366 | G |
|  |  |  | OG11792 | Putative transcriptional regulator (Xre family) |  |  |
|  |  |  | OG11794 | Putative transposase | COG0675 | L |
|  |  |  | OG11849 | Carbamoyl-phosphate synthase , small chain | COG0505 | EF |
|  |  |  | OG11852 | Predicted nucleoside-diphosphate-sugar epimerase | COG0702 | MG |
|  |  |  | OG11856 | Transposase |  |  |
|  |  |  | OG11858 | O-acetylhomoserine (Thiol)-lyase | COG2873 | E |
|  |  |  | OG11860 | Permease | COG0477 | GEPR |
|  |  |  | OG11861 | Permease | COG0477 | GEPR |
|  |  |  | OG11882 | Dipeptidase |  |  |
|  |  |  | OG11887 | Transposase | COG2826 | L |
|  |  |  | OG11891 | Transcriptional regulator | COG1940 | KG |
|  |  |  | OG11894 | Transposase | COG2826 | L |
|  |  |  | OG11897 | Homocysteine S-methyltransferase | COG2040 | E |
|  |  |  | OG11898 | Oligopeptide binding protein | COG4166 | E |
|  |  |  | OG11905 | 5-Methyltetrahydropteroyltriglutamate--homocysteine methyltransferase | COG0620 | E |
|  |  |  | OG11956 | Oligopeptide ABC transporter, substrate binding protein | COG4166 | E |
|  |  |  | OG11972 | 1-Deoxy-D-xylulose 5-phosphate synthase | COG1154 | HI |
|  |  |  | OG11985 | Oligopeptide binding protein | COG4166 | E |
|  |  |  | OG11986 | Peptide ABC transporter substrate-binding protein | COG4166 | E |
|  |  |  | OG12125 | Predicted restriction endonuclease | COG3440 | V |
|  |  |  | OG12131 | Ribose-5-phosphate isomerase | COG0698 | G |
|  |  | Loss | OG10003 | Transposase | COG2963 | L |
|  |  |  | OG10004 | Transposase | COG3464 | L |
|  |  |  | OG10010 | Transposase | COG0675 | L |
|  |  |  | OG10011 | Degenerate transposase | COG3436 | L |
|  |  |  | OG10013 | Putative transposase |  |  |
|  |  |  | OG10014 | Transposase, IS4 family | COG3385 | L |
|  |  |  | OG10163 | Putative Transposase | COG0675 | L |
|  |  |  | OG11366 | dTDP-4-dehydrorhamnose reductase | COG1094 | M |
|  |  |  | OG11367 | dTDP-4-dehydrorhamnose 3,5-epimerase | COG1898 | M |
|  |  |  | OG11374 | LysR family transcriptional regulator | COG0583 | K |
|  |  |  | OG11375 | Laci family transcriptional regulator | COG1609 | K |
|  |  |  | OG11401 | Transposase | COG1943 | L |
|  |  |  | OG11420 | Aldehyde dehydrogenase | COG1012 | C |
|  |  |  | OG11443 | Cell wall-associated hydrolase | COG0791 | M |
|  |  |  | OG11446 | Rec (Receiver) domain signal transduction protein |  |  |
|  |  |  | OG11449 | Pyrroline-5-carboxylate reductase, putative | COG0345 | E |
|  |  |  | OG11450 | Bacterial membrane protein yfho |  |  |
|  |  |  | OG11451 | Transposase |  |  |
|  |  |  | OG11468 | Glucose-1-phosphate thymidylyltransferase | COG1209 | M |
|  |  |  | OG11487 | Arginine deiminase | COG2235 | E |
|  |  |  | OG11488 | Ornithine carbamoyltransferase, catabolic | COG0078 | E |
|  |  |  | OG11490 | 6,7-Dimethyl-8-ribityllumazine synthase | COG0054 | H |
|  |  |  | OG11491 | 3,4-Dihydroxy-2-butanone 4-phosphate synthase | COG0807 | H |
|  |  |  | OG11497 | Cytochrome C551 |  |  |
|  |  |  | OG11499 | Gamma-glutamyl phosphate reductase | COG0014 | E |
|  |  |  | OG11500 | LysR family transcriptional regulator | COG0583 | K |
|  |  |  | OG11531 | LysR family transcriptional regulator | COG0583 | K |
|  |  |  | OG11539 | ABC transporter ATPase | COG1131 | V |
|  |  |  | OG11543 | Carbamate kinase | COG0549 | E |
|  |  |  | OG11547 | Transcriptional regulator | COG0500 | QR |
|  |  |  | OG11550 | Argininosuccinate synthase | COG0137 | E |
|  |  |  | OG11554 | Glutathione red | COG1249 | C |
|  |  |  | OG11556 | Hydrolase, alpha/beta domain protein | COG0657 | I |
|  |  |  | OG11559 | Transposase | COG3335 | L |
|  |  |  | OG11596 | Transposase | COG1943 | L |
|  |  |  | OG11599 | ABC transporter ATP-bindin |  |  |
|  |  |  | OG11600 | von Willebrand factor A |  |  |
|  |  |  | OG11602 | GGDEF domain protein |  |  |
|  |  |  | OG11627 | Transposase | COG2963 | L |
|  |  |  | OG11638 | Oligopeptide binding protein | COG4166 | E |
|  |  |  | OG11643 | Cation transport protein | COG0471 | P |
|  |  |  | OG11658 | Galactose-1-phosphate uridylyltransferase | COG4468 | G |
|  |  |  | OG11659 | Galactokinase | COG0153 | G |
|  |  |  | OG11662 | Lactose phosphotransferase system repressor | COG1349 | KG |
|  |  |  | OG11717 | Oligopeptide ABC transporter substrate-binding protein | COG4166 | E |
|  |  |  | OG11813 | Haloacid dehalogenase | COG0561 | R |
|  |  |  | OG11818 | Branched-chain amino acid ABC transporter substrate-binding protein | COG0683 | E |

**Supplementary table 5. Orthologous genes gained/lost in the *lactis* lineage*.***

| 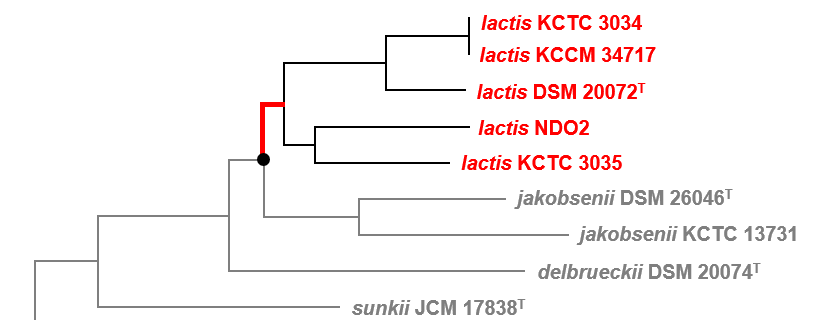 | | | | | | |
| --- | --- | --- | --- | --- | --- | --- |
| **Evolution path** | | **Gain/**  **loss** | **Orthologous gene_Fam** | **Annotation** | **COG No.** | **COG cat.** |
| **From** | **To** |  |  |  |  |  |
| Common ancestor of *lactis-jakobsenii* lineage | *lactis* lineage | Gain | OG10001 | Transposase | COG3547 | L |
|  |  |  | OG10002 | Transposase | COG3328 | L |
|  |  |  | OG10003 | Transposase | COG2963 | L |
|  |  |  | OG10007 | Transposase |  |  |
|  |  |  | OG10013 | Transposase |  |  |
|  |  |  | OG10030 | Transposase | COG0675 | L |
|  |  |  | OG10050 | D-aminopeptidase | COG2362 | E |
|  |  |  | OG11183 | Type I restriction-modification system, restriction subunit | COG0610 | V |
|  |  |  | OG11337 | Serine/threonine protein kinase | COG0515 | RTKL |
|  |  |  | OG11384 | Lac repressor | COG1609 | K |
|  |  |  | OG11414 | Permease | COG2814 | G |
|  |  |  | OG11421 | Beta-galactosidase | COG3250 | G |
|  |  |  | OG11452 | Transposase A | COG2452 | L |
|  |  |  | OG11503 | HNH endonuclease | COG1403 | V |
|  |  |  | OG11504 | PTS system, mannitol-specific IIBC component | COG2213 | G |
|  |  |  | OG11511 | Antitoxin HicB |  |  |
|  |  |  | OG11516 | Peptide binding protein | COG4166 | E |
|  |  |  | OG11569 | Toxin HicA |  |  |
|  |  |  | OG11603 | PTS system, mannitol-specific IIA component | COG4668 | G |
|  |  |  | OG11604 | Phosphoglycerate mutase | COG0406 | G |
|  |  |  | OG11611 | Type III restriction enzyme, res subunit | COG0827 | L |
|  |  |  | OG11619 | Transcription regulator | COG0583 | K |
|  |  |  | OG11652 | Alpha/beta superfamily hydrolase | COG1073 | R |
|  |  |  | OG11653 | ATPase | COG1373 | R |
|  |  |  | OG11661 | PTS family lactose porter, IICB component | COG1455 | G |
|  |  |  | OG11667 | Cold-shock DNA-binding protein family | COG1278 | K |
|  |  |  | OG11719 | 3-Dehydroquinate dehydratase | COG0710 | E |
|  |  |  | OG11720 | Phosphoenolpyruvate-dependent sugar phosphotransferase system EIIA, lactose specific | COG1447 | G |
|  |  |  | OG11726 | Oligopeptide ABC superfamily ATP binding cassette transporter | COG4166 | E |
|  |  |  | OG11728 | Dihydroxyacetone kinase, phosphotransfer subunit | COG3412 | S |
|  |  |  | OG11729 | Dihydroxyacetone kinase, L subunit | COG2376 | G |
|  |  |  | OG11730 | Dihydroxyacetone kinase, DhaK subunit | COG2376 | G |
|  |  |  | OG11748 | Diguanylate cyclase domain protein | COG2199 | T |
|  |  |  | OG11804 | Transposase A | COG2452 | L |
|  |  |  | OG11806 | Cytosine-specific methyltransferase | COG0270 | L |
|  |  |  | OG11837 | 5-Methyltetrahydropteroyltriglutamate--homocysteine S-methyltransferase | COG0620 | E |
|  |  |  | OG11922 | Phage/plasmid primase | COG3378 | R |
|  |  |  | OG11926 | SOS regulatory protein LexA | COG1974 | KT |
|  |  |  | OG11927 | Bacitracin transport ATP binding cassette transporter, ABC protein |  |  |
|  |  |  | OG11942 | Stress response regulator Gls24 | COG1302 | S |
|  |  |  | OG12163 | Cytosine-specific methyltransferase | COG0270 | L |
|  |  | Loss | OG10014 | Transposase, IS4 family | COG3385 | L |
|  |  |  | OG11457 | Putative membrane protein |  |  |

**Supplementary table 6. Orthologous genes gained/lost in the *jakobsenii* lineage*.***

| 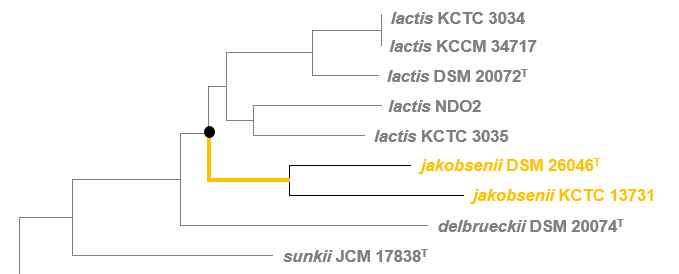 | | | | | | |
| --- | --- | --- | --- | --- | --- | --- |
| **Evolution path** | | **Gain/**  **loss** | **Orthologous gene_Fam** | **Annotation** | **COG No.** | **COG cat.** |
| **From** | **To** |  |  |  |  |  |
| Common ancestor of *lactis-jakobsenii* lineage | *jakobsenii* lineage | Gain | OG10014 | Transposase, IS4 family | COG3385 | L |
|  |  |  | OG10016 | Putative phosphoglycerol transferase | COG1368 | M |
|  |  |  | OG10049 | UmuC-like DNA-repair protein | COG0389 | L |
|  |  |  | OG10071 | Dipeptidase | COG4690 | E |
|  |  |  | OG10160 | Hypothetical protein |  |  |
|  |  |  | OG11072 | Predicted membrane protein |  |  |
|  |  |  | OG11405 | ABC transporter ATP binding and permease protein | COG1132 | V |
|  |  |  | OG11450 | Bacterial membrane protein yfho |  |  |
|  |  |  | OG11456 | Transcriptional regulator, xre family |  |  |
|  |  |  | OG11463 | DNA invertase | COG2452 | L |
|  |  |  | OG11508 | ABC-type multidrug transport system, ATPase component | COG4586 | R |
|  |  |  | OG11515 | Histidinol-phosphatase | COG2199 | T |
|  |  |  | OG11614 | Glycosyltransferase | COG1442 | M |
|  |  |  | OG11640 | Succinate dehydrogenase | COG1053 | C |
|  |  |  | OG11642 | Glycosyltransferase related enzyme | COG0463 | M |
|  |  |  | OG11650 | CRISPR-associated endonuclease Cas1 | COG1518 | L |
|  |  |  | OG11673 | Transcriptional regulator |  |  |
|  |  |  | OG11710 | CRISPR-associated endoribonuclease Cas2 | COG3512 | S |
|  |  |  | OG11711 | CRISPR-associated protein |  |  |
|  |  |  | OG11733 | ABC transporter, ATP-binding protein | COG1132 | V |
|  |  |  | OG11809 | Extracellular zinc metalloproteinase |  |  |
|  |  |  | OG11909 | Transposase IS607 family protein | COG0675 | L |
|  |  |  | OG11910 | ABC antimicrobial peptide transporter ATPase | COG1136 | V |
|  |  |  | OG11993 | Transposase |  |  |
|  |  |  | OG11998 | Dinucleotide-utilizing protein | COG0476 | H |
|  |  |  | OG12085 | Putative transcriptional regulator | COG2865 | K |
|  |  |  | OG12105 | XRE family transcriptional regulator | COG1396 | K |
|  |  |  | OG12143 | Branched-chain amino acid transport protein AzlD | COG4392 | S |
|  |  |  | OG12144 | LIV-E family branched chain amino acid exporter AzlC | COG1296 | E |
|  |  |  | OG12147 | Transporter protein | COG0477 | GEPR |
|  |  |  | OG12148 | HesA/MoeB/ThiF family protein | COG0476 | H |
|  |  |  | OG12159 | FMN-binding domain protein | COG3976 | S |
|  |  |  | OG12322 | Transposase |  |  |
|  |  |  | OG12324 | Replication protein |  |  |
|  |  |  | OG12325 | Branched-chain amino acid transport system II carrier protein | COG1114 | E |
|  |  |  | OG12326 | Branched-chain amino acid transporter | COG1114 | E |
|  |  |  | OG12331 | Oligopeptide binding protein | COG4166 | E |
|  |  |  | OG12339 | Diguanylate cyclase | COG2199 | T |
|  |  |  | OG12343 | Transposase | COG2452 | L |
|  |  |  | OG12344 | Putative transcriptional regulator |  |  |
|  |  | Loss | OG10003 | Transposase | COG2963 | L |
|  |  |  | OG10056 | Predicted transcriptional regulator | COG0789 | K |
|  |  |  | OG10614 | Diguanylate cyclase (GGDEF) domain protein | COG2199 | T |
|  |  |  | OG11237 | Permease | COG0477 | GEPR |
|  |  |  | OG11270 | Riboflavin biosynthesis protein RibD | COG1985 | H |
|  |  |  | OG11281 | ABC transporter, ATP-binding/permease protein | COG1132 | V |
|  |  |  | OG11300 | ABC transporter, ATP-binding protein | COG1122 | P |
|  |  |  | OG11327 | Putative anti-SigV factor |  |  |
|  |  |  | OG11353 | Lactose permease | COG2211 | G |
|  |  |  | OG11379 | ATP synthase subunit c | COG0636 | C |
|  |  |  | OG11381 | ABC transporter, permease protein | COG0619 | P |
|  |  |  | OG11382 | Signal transduction histidine kinase, LytS |  |  |
|  |  |  | OG11416 | Putative membrane protein |  |  |
|  |  |  | OG11429 | Putative biotin carboxylase |  |  |
|  |  |  | OG11430 | Proline dipeptidase | COG0006 | E |
|  |  |  | OG11431 | Diguanylate cyclase (GGDEF) domain protein |  |  |
|  |  |  | OG11432 | Fe-S cluster assembly ABC-type transport system, permease component | COG0719 | O |
|  |  |  | PG11434 | Dipeptidase | COG4690 | E |
|  |  |  | OG11435 | Glycerophosphoryl diester phosphodiesterase | COG0584 | C |
|  |  |  | OG11442 | Transposase |  |  |
|  |  |  | OG11460 | Putative signal transduction protein |  |  |
|  |  |  | OG11558 | Antibiotic biosynthesis monooxygenase subfamily | COG1359 | S |
|  |  |  | OG11609 | Riboflavin synthase subunit alpha | COG0307 | H |
|  |  |  | OG11625 | Transposase DDE domain |  |  |
|  |  |  | OG11643 | Cation transport protein | COG0471 | P |
|  |  |  | OG11724 | Vitamin-B12 independent methionine synthase | COG0620 | E |
|  |  |  | OG11725 | Oligopeptide ABC superfamily ATP binding cassette transporter |  |  |
|  |  |  | OG11839 | Transglycosylase associated protein | COG2261 | S |

**Supplementary table 7. Orthologous genes gained/lost in the *delbrueckii* lineage*.***

| 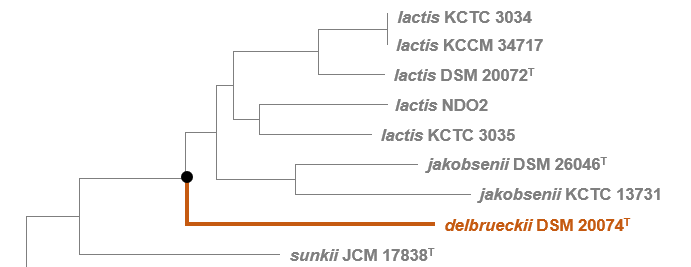 | | | | | | |
| --- | --- | --- | --- | --- | --- | --- |
| **Evolution path** | | **Gain/**  **loss** | **Orthologous gene_Fam** | **Annotation** | **COG No.** | **COG cat.** |
| **From** | **To** |  |  |  |  |  |
| Common ancestor of *lactis-jakobsenii-delbrueckii-sunkii* lineage | *delbrueckii* lineage | Gain | OG10000 | Transposase | COG2826 | L |
|  |  |  | OG10001 | Transposase | COG3547 | L |
|  |  |  | OG10003 | Transposase | COG2963 | L |
|  |  |  | OG10004 | Transposase | COG3464 | L |
|  |  |  | OG10013 | Putative transposase |  |  |
|  |  |  | OG10032 | Ornithine decarboxylase | COG1982 | E |
|  |  |  | OG10036 | Predicted membrane protein | COG3274 | S |
|  |  |  | OG10057 | Transport permease protein | COG0842 | V |
|  |  |  | OG10067 | Enolase | COG0148 | G |
|  |  |  | OG10076 | Permease | COG0477 | GEPR |
|  |  |  | OG10089 | Glutamine amidotransferase | COG2071 | R |
|  |  |  | OG10090 | Putative chloride channel protein | COG0038 | P |
|  |  |  | OG10101 | Transcriptional regulator, AraC family | COG2207 | K |
|  |  |  | OG10103 | Glutamine--fructose-6-phosphate aminotransferase [isomerizing] | COG0449 | M |
|  |  |  | OG10104 | Glutamate racemase | COG0796 | M |
|  |  |  | OG10105 | Dipeptidase | COG4690 | E |
|  |  |  | OG10107 | Uridine kinase | COG0572 | F |
|  |  |  | OG10108 | Glutamine ABC transporter ATPase component | COG1126 | E |
|  |  |  | OG10109 | Glutamine ABC transporter, permease protein | COG0765 | E |
|  |  |  | OG10110 | Glutamine ABC transporter, permease protein | COG0765 | E |
|  |  |  | OG10159 | Oxidoreductase | COG0778 | C |
|  |  |  | OG10162 | Cellobiose-specific PTS system IIC component | COG1455 | G |
|  |  |  | OG10349 | ABC transporter ATP-binding protein | COG0488 | R |
|  |  |  | OG10350 | Membrane protein | COG0670 | R |
|  |  |  | OG10351 | Glutamyl aminopeptidase | COG1363 | G |
|  |  |  | OG10528 | D-Hydroxyisocaproate dehydrogenase | COG1052 | CHR |
|  |  |  | OG10689 | Putative helicase | COG1201 | R |
|  |  |  | OG10857 | Alpha-amylase | COG0366 | G |
|  |  |  | OG10991 | Membrane protein | COG3759 | S |
|  |  |  | OG10992 | Phosphoenolpyruvate carboxylase | COG2352 | C |
|  |  |  | OG10993 | Glutamine ABC transporter, glutamine-binding protein | COG0834 | ET |
|  |  |  | OG11130 | Putative hydrolase (NUDIX family) | COG2816 | L |
|  |  |  | OG11270 | Riboflavin biosynthesis protein RibD | COG1985 | H |
|  |  |  | OG11293 | Maltose O-acetyltransferase |  |  |
|  |  |  | OG11337 | Serine/threonine protein kinase | COG0515 | RTKL |
|  |  |  | OG11347 | ABC transporter, ATP-binding protein | COG1132 | V |
|  |  |  | OG11374 | LysR family transcriptional regulator | COG0583 | K |
|  |  |  | OG11414 | Permease | COG2814 | G |
|  |  |  | OG11448 | Maltose phosphorylase | COG1554 | G |
|  |  |  | OG11453 | Transposase |  |  |
|  |  |  | OG11467 | Transposase | COG2826 | L |
|  |  |  | OG11478 | Predicted membrane protein |  |  |
|  |  |  | OG11503 | HNH endonuclease | COG1403 | V |
|  |  |  | OG11505 | Myosin-crossreactive antigen | COG4716 | S |
|  |  |  | OG11511 | Antitoxin HicB |  |  |
|  |  |  | OG11516 | Peptide binding protein | COG4166 | E |
|  |  |  | OG11517 | Sensory box/GGDEF family protein | COG2200 | T |
|  |  |  | OG11525 | Signal transduction diguanylate cyclase | COG2199 | T |
|  |  |  | OG11540 | Fumarate reductase, flavoprotein subunit, N-terminally truncated | COG3976 | S |
|  |  |  | OG11559 | Transposase | COG3335 | L |
|  |  |  | OG11561 | Glycine betaine/L-proline ABC superfamily ATP binding cassette transporter, binding protein | COG1732 | M |
|  |  |  | OG11576 | Alpha/beta hydrolase superfamily protein | COG4814 | R |
|  |  |  | OG11579 | Oligopeptide ABC transporter, substrate binding protein | COG4166 | E |
|  |  |  | OG11585 | Phosphotransferase with a nitrogenous group as acceptor | COG2141 | C |
|  |  |  | OG11594 | Transposase | COG3328 | L |
|  |  |  | OG11604 | Phosphoglycerate mutase | COG0406 | G |
|  |  |  | OG11620 | Peptide binding protein | COG4166 | E |
|  |  |  | OG11627 | Transposase | COG2963 | L |
|  |  |  | OG11641 | Transposase |  |  |
|  |  |  | OG11664 | Binding-protein-dependent transport systems inner membrane component | COG1174 | E |
|  |  |  | OG11665 | Glycine/betaine/carnitine/choline ABC superfamily ATP binding cassette transporter, membrane protein | COG1174 | E |
|  |  |  | OG11633 | Glycine betaine/L-proline ABC superfamily ATP binding cassette transporter, ABC protein | COG1125 | E |
|  |  |  | OG11673 | Transcriptional regulator |  |  |
|  |  |  | OG11691 | Pyruvate,water dikinase | COG0574 | G |
|  |  |  | OG11723 | Multidrug resistance ABC superfamily ATP binding cassette transporter ATP-binding and permease |  |  |
|  |  |  | OG11757 | Putative ABC transporter, ATP-binding protein | COG1116 | P |
|  |  |  | OG11764 | N-6 DNA methylase | COG0286 | V |
|  |  |  | OG11787 | EAL domain protein |  |  |
|  |  |  | OG11791 | Alpha-amylase | COG0366 | G |
|  |  |  | OG11808 | Lactose transport regulator |  |  |
|  |  |  | OG11836 | Permease IIC component | COG1455 | G |
|  |  |  | OG11837 | 5-Methyltetrahydropteroyltriglutamate--homocysteine S-methyltransferase | COG0620 | E |
|  |  |  | OG11859 | Hydrolase | COG0561 | R |
|  |  |  | OG11879 | SNF2-related domain:Helicase, C-terminal:SWIM Zn-finger | COG0553 | KL |
|  |  |  | OG11901 | Prismane protein | COG1073 | R |
|  |  |  | OG11929 | Glycosyl transferase | COG0463 | M |
|  |  |  | OG11931 | ROK family protein | COG1940 | KG |
|  |  |  | OG11932 | Alpha-amylase |  |  |
|  |  |  | OG11934 | Oxidoreductase (Aldo/keto reductase family) | COG0656 | R |
|  |  |  | OG11942 | Stress response regulator Gls24 | COG1302 | S |
|  |  |  | OG11943 | Eps operon transcriptional regulator EpsIIA | COG1316 | K |
|  |  |  | OG11944 | Polymerase for repeating unit of receptor polysaccharide |  |  |
|  |  |  | OG11945 | Eps10P |  |  |
|  |  |  | OG11973 | Permease | COG0477 | GEPR |
|  |  |  | OG11992 | Transposase |  |  |
|  |  |  | OG12003 | Topology modulation protein | COG0563 | F |
|  |  |  | OG12014 | Putative membrane protein |  |  |
|  |  |  | OG12015 | Bacterial regulatory s, tetR family protein |  |  |
|  |  |  | OG12016 | Bacterial regulatory s, tetR family protein |  |  |
|  |  |  | OG12017 | Pyridoxal-dependent decarboxylase | COG0076 | E |
|  |  |  | OG12018 | Amino acid adenylation domain protein | COG1020 | Q |
|  |  |  | OG12019 | AMP-binding enzyme family protein |  |  |
|  |  |  | OG12020 | Trp repressor binding protein |  |  |
|  |  |  | OG12029 | ABC superfamily ATP binding cassette transporter | COG1132 | V |
|  |  |  | OG12030 | PTS family protein, cellobiose-specific IIA component | COG1447 | G |
|  |  |  | OG12033 | Oligopeptide ABC superfamily ATP binding cassette transporter, binding protein | COG4166 | E |
|  |  |  | OG12037 | Type I restriction-modification system specificity subunit |  |  |
|  |  |  | OG12038 | Transposase | COG3436 | L |
|  |  |  | OG12039 | Rib/alpha-like repeat protein |  |  |
|  |  |  | OG12040 | Capsule biosynthesis protein CapC |  |  |
|  |  |  | OG12041 | Glycosyl transferase | COG0463 | M |
|  |  |  | OG12079 | Restriction endonuclease family protein | COG1715 | V |
|  |  |  | OG12098 | Transferase for other substituted phosphate groups | COG3475 | M |
|  |  |  | OG12103 | Riboflavin synthase alpha chain | COG0307 | H |
|  |  |  | OG12108 | Oligopeptide binding protein |  |  |
|  |  |  | OG12132 | Transposase |  |  |
|  |  |  | OG12134 | Diguanylate cyclase (GGDEF) domain protein | COG2199 | T |
|  |  |  | OG12152 | ABC transporter, ATP-binding protein | COG1116 | P |
|  |  |  | OG12191 | Type I site-specific deoxyribonuclease, HsdR family | COG0610 | V |
|  |  |  | OG12192 | Integrase | COG0582 | L |
|  |  |  | OG12193 | N-6 DNA methylase | COG0286 | V |
|  |  |  | OG12197 | Surface antigen repeat protein |  |  |
|  |  |  | OG12198 | Glucansucrase |  |  |
|  |  |  | OG12199 | Glycosyl transferase | COG0463 | M |
|  |  |  | OG12200 | Capsular biosynthesis protein |  |  |
|  |  |  | OG12201 | Hexosyltransferase | COG0438 | M |
|  |  |  | OG12202 | Hypothetical protein |  |  |
|  |  |  | OG12203 | Alcohol dehydrogenase GroES domain protein | COG0604 | CR |
|  |  |  | OG12342 | Transposase | COG0675 | L |
|  |  |  | OG12366 | Pnp/Udp family phosphorylase |  |  |
|  |  |  | OG12376 | Cyclic diguanylate phosphodiesterase (EAL) domain protein | COG2200 | T |
|  |  |  | OG12377 | Transposase | COG3547 | L |
|  |  |  | OG12378 | Transposase | COG2801 | L |
|  |  |  | OG12380 | Acetyltransferase | COG0110 | R |
|  |  |  | OG12383 | Helicase conserved C-terminal domain protein | COG0514 | L |
|  |  |  | OG12386 | Transposase |  |  |
|  |  |  | OG12387 | Histidine kinase | COG0642 | T |
|  |  |  | OG12391 | Carnitine transporter | COG1292 | M |
|  |  |  | OG12392 | Cholinephosphate cytidylyltransferase | COG4750 | M |
|  |  |  | OG12393 | Phosphoenolpyruvate mutase | COG0510 | M |
|  |  |  | OG12400 | Transcriptional regulator | COG1959 | K |
|  |  |  | OG12402 | Putative peptidoglycan binding domain protein | COG3409 | M |
|  |  |  | OG12911 | Transposase, IS30 family | COG2826 | L |
|  |  |  | OG12915 | Branched-chain amino acid transport system II carrier protein | COG1114 | E |
|  |  |  | OG12916 | Branched-chain amino acid transport system carrier protein | COG1114 | E |
|  |  |  | OG12917 | DNA alkylation repair enzyme | COG4912 | L |
|  |  |  | OG12918 | Transcriptional regulator, AsnC family | COG1522 | K |
|  |  |  | OG12920 | Transposase, IS116/IS110/IS902 family |  |  |
|  |  |  | OG12921 | Transposase | COG3547 | L |
|  |  |  | OG12922 | Peptidase M16 | COG0612 | R |
|  |  |  | OG12923 | ATP synthase subunit c | COG0636 | C |
|  |  |  | OG12925 | Transposase | COG2452 | L |
|  |  |  | OG12930 | Formate/nitrite family of transporter | COG2116 | P |
|  |  |  | OG12937 | Transposase | COG2826 | L |
|  |  |  | OG12940 | Multidrug transporter MatE | COG0534 | V |
|  |  |  | OG12944 | Transposase A | COG2452 | L |
|  |  |  | OG12945 | Restriction endonuclease subunit S | COG0732 | V |
|  |  |  | OG12948 | Type I restriction-modification system specificity subunit |  |  |
|  |  |  | OG12951 | RNA polymerase sigma factor, sigma-70 family protein |  |  |
|  |  |  | OG12952 | S9A/B/C family peptidase | COG1506 | E |
|  |  |  | OG12954 | ABC superfamily ATP binding cassette transporter permease subunit |  |  |
|  |  |  | OG12955 | ABC transporter permease |  |  |
|  |  |  | OG12956 | Glycosyltransferase | COG1215 | M |
|  |  |  | OG12959 | Glycosyl hydrolase, family 1 | COG2723 | G |
|  |  |  | OG12961 | Transposase |  |  |
|  |  |  | OG12962 | Transposase |  |  |
|  |  |  | OG12971 | Phosphoribosylformylglycinamidine synthase subunit PurL | COG0046 | F |
|  |  |  | OG12974 | MerR regulatory family protein | COG2452 | L |
|  |  |  | OG12976 | Transposase | COG2826 | L |
|  |  |  | OG12978 | DNA-binding protein | COG0776 | L |
|  |  |  | OG12979 | Transposase | COG2826 | L |
|  |  |  | OG12981 | Transposase | COG3464 | L |
|  |  |  | OG12982 | Peptidase m20a dipeptidase |  |  |
|  |  |  | OG12986 | Transposase | COG2452 | L |
|  |  |  | OG12989 | GMP synthase-Glutamine amidotransferase domain | COG0518 | F |
|  |  |  | OG12990 | Glutamine ABC superfamily ATP binding cassette transporter, membrane protein | COG0765 | E |
|  |  |  | OG12991 | Transposase | COG2826 | L |
|  |  |  | OG12992 | Transposase, IS30 family | COG2826 | L |
|  |  |  | OG12993 | Transposase | COG2826 | L |
|  |  |  | OG13000 | Cupin domain protein | COG0662 | G |
|  |  |  | OG13001 | Transposase |  |  |
|  |  |  | OG13005 | Membrane protein | COG2261 | S |
|  |  |  | OG13006 | Transposase |  |  |
|  |  |  | OG13008 | Transposase | COG0675 | L |
|  |  |  | OG13011 | Transposase | COG3335 | L |
|  |  |  | OG13013 | DNA adenine methylase Dam | COG0338 | L |
|  |  |  | OG13020 | Transposase DDE domain |  |  |
|  |  |  | OG13021 | Transposase |  |  |
|  |  |  | OG13022 | Oligopeptide ABC superfamily ATP binding cassette transporter, binding protein | COG4166 | E |
|  |  |  | OG13023 | Oligopeptide ABC superfamily ATP binding cassette transporter | COG4166 | E |
|  |  |  | OG13024 | Transposase | COG2826 | L |
|  |  |  | OG13025 | Transposase, IS30 family | COG2826 | L |
|  |  |  | OG13026 | Transposase, IS605 OrfB family |  |  |
|  |  |  | OG13029 | Aromatic amino acid aminotransferase domain protein | COG0436 | E |
|  |  |  | OG13031 | Uridine kinase (Fragmented) | COG0572 | F |
|  |  |  | OG13032 | Uridine kinase (Fragmented) | COG0572 | F |
|  |  |  | OG13033 | Glutamine ABC transporter substrate-binding protein | COG0834 | ET |
|  |  | Loss | OG10005 | Peptide binding protein | COG4166 | E |
|  |  |  | OG10011 | Degenerate transposase | COG3436 | L |
|  |  |  | OG10014 | Transposase, IS4 family | COG3385 | L |
|  |  |  | OG10015 | Transposase | COG3464 | L |
|  |  |  | OG10074 | Homoserine O-succinyltransferase | COG1897 | E |
|  |  |  | OG10082 | ABC transporter, ATP-binding/permease protein | COG1132 | V |
|  |  |  | OG10083 | Phosphoglycerate dehydrogenase | COG0111 | HE |
|  |  |  | OG10102 | Carbamoyl-phosphate synthase |  |  |
|  |  |  | OG10163 | Putative Transposase | COG0675 | L |
|  |  |  | OG10201 | Permease | COG0477 | GEPR |
|  |  |  | OG10957 | Adenosylcobalamin-dependent ribonucleoside-triphosphate reductase | COG1372 | L |
|  |  |  | OG10959 | Succinate dehydrogenase | COG0169 | E |
|  |  |  | OG10960 | Protease HtpX homolog | COG0501 | O |
|  |  |  | OG10961 | LemA-like protein | COG1704 | S |
|  |  |  | OG10962 | Putative permease | COG0471 | P |
|  |  |  | OG11054 | ABC transporter, ATP-binding protein | COG1122 | P |
|  |  |  | OG11072 | Predicted membrane protein |  |  |
|  |  |  | OG11106 | Permease | COG0477 | GEPR |
|  |  |  | OG11108 | Hypothetical protein |  |  |
|  |  |  | OG11132 | Mannitol operon transcriptional regulator |  |  |
|  |  |  | OG11179 | Phosphoserine aminotransferase | COG1932 | HE |
|  |  |  | OG11219 | Adenosine deaminase | COG1816 | F |
|  |  |  | OG11237 | Permease | COG0477 | GEPR |
|  |  |  | OG11274 | Transposase |  |  |
|  |  |  | OG11281 | ABC transporter, ATP-binding/permease protein | COG1132 | V |
|  |  |  | OG11282 | Pyridoxal kinase | COG2240 | H |
|  |  |  | OG11283 | Predicted membrane protein | COG4720 | S |
|  |  |  | OG11286 | Putative transcriptional regulator (AraC family) | COG2207 | K |
|  |  |  | OG11288 | O-Acetylhomoserine aminocarboxypropyltransferase | COG2873 | E |
|  |  |  | OG11291 | Mrr restriction system protein | COG1715 | V |
|  |  |  | OG11294 | Mannitol-1-phosphate 5-dehydrogenase | COG0246 | G |
|  |  |  | OG11304 | Succinate dehydrogenase | COG3279 | KT |
|  |  |  | OG11306 | Acyltransferase | COG1247 | M |
|  |  |  | OG11309 | S-Methyltransferase | COG0350 | L |
|  |  |  | OG11311 | Aspartokinase | COG0527 | E |
|  |  |  | OG11313 | Homoserine dehydrogenase | COG0460 | E |
|  |  |  | OG11323 | ABC-type cobalt transport system, permease component CbiQ related transporter | COG0619 | P |
|  |  |  | OG11340 | Carbonate dehydratase | COG3338 | P |
|  |  |  | OG11353 | Lactose permease | COG2211 | G |
|  |  |  | OG11356 | Putative secreted 5'-nucleotidase | COG0737 | F |
|  |  |  | OG11359 | Cysteine synthase | COG0031 | E |
|  |  |  | OG11360 | Transposase |  |  |
|  |  |  | OG11365 | EpsIIC, Putative glycosyltransferase | COG2148 | M |
|  |  |  | OG11370 | LPXTG-motif cell wall anchor domain protein | COG1114 | E |
|  |  |  | OG11379 | ATP synthase subunit c | COG0636 | C |
|  |  |  | OG11381 | ABC transporter, permease protein | COG0619 | P |
|  |  |  | OG11382 | Signal transduction histidine kinase, LytS |  |  |
|  |  |  | OG11390 | Biotin carboxylase |  |  |
|  |  |  | OG11401 | Transposase family protein A | COG1943 | L |
|  |  |  | OG11402 | Methylase |  |  |
|  |  |  | OG11411 | Transport permease protein | COG0842 | V |
|  |  |  | OG11412 | ABC transporter, ATP-binding protein | COG1131 | V |
|  |  |  | OG11418 | Putative transcriptional regulator (LysR family) | COG0583 | K |
|  |  |  | OG11422 | Transcription regulator | COG1316 | K |
|  |  |  | OG11426 | Putative transcriptional regulator | COG2207 | K |
|  |  |  | OG11427 | Shikimate kinase | COG0703 | E |
|  |  |  | OG11429 | Putative biotin carboxylase |  |  |
|  |  |  | OG11430 | Proline dipeptidase | COG0006 | E |
|  |  |  | OG11434 | Dipeptidase | COG4690 | E |
|  |  |  | OG11435 | Glycerophosphoryl diester phosphodiesterase | COG0584 | C |
|  |  |  | OG11436 | Glycosyltransferase | COG0438 | M |
|  |  |  | OG11451 | Transposase |  |  |
|  |  |  | OG11460 | Putative signal transduction protein |  |  |
|  |  |  | OG11476 | Predicted integral membrane protein |  |  |
|  |  |  | OG11477 | EpsIIG, Putative glycosyltransferase | COG1216 | R |
|  |  |  | OG11533 | Putative membrane protein |  |  |
|  |  |  | OG11534 | ABC transporter, ATP-binding protein | COG1131 | V |
|  |  |  | OG11536 | MutG family lantibiotic protection ABC superfamily ATP binding cassette transporter permease subunit |  |  |
|  |  |  | OG11544 | Phosphoenolpyruvate-dependent sugar phosphotransferase system eiiABC, trhalose specific | COG1263 | G |
|  |  |  | OG11545 | Trehalose operon transcriptional repressor | COG2188 | K |
|  |  |  | OG11546 | Alpha,alpha-phosphotrehalase (Gh13) | COG0366 | G |
|  |  |  | OG11549 | Integrase/recombinase |  |  |
|  |  |  | OG11573 | Pyruvate/2-oxoglutarate dehydrogenase complex, dihydrolipoamide dehydrogenase (E3) component | COG1249 | C |
|  |  |  | OG11590 | Brp/Blh family beta-carotene 15,15'-monooxygenase |  |  |
|  |  |  | OG11592 | Putative membrane protein |  |  |
|  |  |  | OG11593 | P-loop ATPase |  |  |
|  |  |  | OG11597 | DNA starvation/stationary phase protection protein Dps | COG0783 | P |
|  |  |  | OG11598 | Formate/nitrite family of transporter | COG2116 | P |
|  |  |  | OG11644 | Putative secreted protein |  |  |
|  |  |  | OG11646 | Chromosome partitioning protein ParA | COG1192 | D |
|  |  |  | OG11658 | Galactose-1-phosphate uridylyltransferase | COG4468 | G |
|  |  |  | OG11659 | Galactokinase | COG0153 | G |
|  |  |  | OG11662 | Lactose phosphotransferase system repressor | COG1349 | KG |
|  |  |  | OG11813 | Haloacid dehalogenase | COG0561 | R |
|  |  |  | OG11839 | Transglycosylase associated protein | COG2261 | S |

**Supplementary table 8. Orthologous genes gained/lost in the *sunkii* lineage.**

| 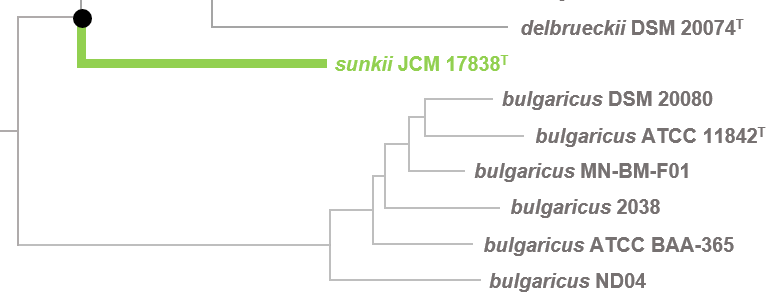 | | | | | | |
| --- | --- | --- | --- | --- | --- | --- |
| **Evolution path** | | **Gain/**  **loss** | **Orthologous gene_Fam** | **Annotation** | **COG No.** | **COG cat.** |
| **From** | **To** |  |  |  |  |  |
| Common ancestor of *lactis-jakobsenii-delbrueckii-sunkii* lineages | *sunkii* lineage | Gain | OG10005 | Peptide binding protein | COG4166 | E |
|  |  |  | OG10010 | Transposase ORF_B | COG0675 | L |
|  |  |  | OG10014 | Transposase, IS4 family | COG3385 | L |
|  |  |  | OG10049 | UmuC-like DNA-repair protein | COG0389 | L |
|  |  |  | OG10084 | Signal peptidase I | COG0681 | U |
|  |  |  | OG10088 | EpsIIE, UDP-galactopyranose mutase | COG0562 | M |
|  |  |  | OG10094 | Transcriptional regulator and fructokinase | COG1940 | KG |
|  |  |  | OG10118 | Putative signal transduction protein | COG2199 | T |
|  |  |  | OG10163 | Putative Transposase | COG0675 | L |
|  |  |  | OG10959 | Succinate dehydrogenase | COG0169 | E |
|  |  |  | OG11279 | Transposase ORF_A | COG2452 | L |
|  |  |  | OG11397 | ABC sugar transporter ATPase | COG3839 | G |
|  |  |  | OG11401 | Transposase family protein A | COG1943 | L |
|  |  |  | OG11405 | ABC transporter ATP binding and permease protein | COG1132 | V |
|  |  |  | OG11425 | Plasmid mobilization system relaxase | COG1053 | C |
|  |  |  | OG11447 | Brp/Blh family beta-carotene 15,15'-monooxygenase |  |  |
|  |  |  | OG11450 | Bacterial membrane protein yfho |  |  |
|  |  |  | OG11504 | PTS system, mannitol-specific IIBC component | COG2213 | G |
|  |  |  | OG11517 | Sensory box/GGDEF family protein | COG2200 | T |
|  |  |  | OG11524 | Permease | COG0477 | GEPR |
|  |  |  | OG11530 | LPS biosynthesis protein | COG3475 | M |
|  |  |  | OG11570 | Transposase | COG0675 | L |
|  |  |  | OG11574 | Transposase | COG3547 | L |
|  |  |  | OG11584 | EpsIIL, Putative polysaccharide repeat unit transport protein | COG2244 | R |
|  |  |  | OG11603 | PTS system, mannitol-specific IIA component | COG4668 | G |
|  |  |  | OG11612 | Transcriptional regulator, xre family |  |  |
|  |  |  | OG11618 | Signal transduction diguanylate cyclase |  |  |
|  |  |  | OG11619 | Transcription regulator | COG0583 | K |
|  |  |  | OG11640 | Succinate dehydrogenase | COG1053 | C |
|  |  |  | OG11650 | CRISPR-associated endonuclease Cas1 | COG1518 | L |
|  |  |  | OG11652 | Alpha/beta superfamily hydrolase | COG1073 | R |
|  |  |  | OG11653 | ATPase | COG1373 | R |
|  |  |  | OG11681 | Putative ABC transporter, ATP-binding protein | COG1116 | P |
|  |  |  | OG11695 | Galactofuranosyltransferase |  |  |
|  |  |  | OG11715 | Sortase |  |  |
|  |  |  | OG11718 | Transposase | COG0675 | L |
|  |  |  | OG11719 | 3-Dehydroquinate dehydratase | COG0710 | E |
|  |  |  | OG11726 | Oligopeptide ABC superfamily ATP binding cassette transporter | COG4166 | E |
|  |  |  | OG11728 | Dihydroxyacetone kinase, phosphotransfer subunit | COG3412 | S |
|  |  |  | OG11729 | Dihydroxyacetone kinase, L subunit | COG2376 | G |
|  |  |  | OG11730 | Dihydroxyacetone kinase, DhaK subunit | COG2376 | G |
|  |  |  | OG11733 | ABC transporter, ATP-binding protein | COG1132 | V |
|  |  |  | OG11748 | Diguanylate cyclase domain protein | COG2199 | T |
|  |  |  | OG11779 | Transposase |  |  |
|  |  |  | OG11804 | Transposase A | COG2452 | L |
|  |  |  | OG11810 | ABC superfamily ATP binding cassette transporter, permease | COG0395 | G |
|  |  |  | OG11822 | XRE family transcriptional regulator |  |  |
|  |  |  | OG11824 | Wzy |  |  |
|  |  |  | OG11838 | IS1272 transposase | COG3666 | L |
|  |  |  | OG11842 | Transcription regulator | COG0583 | K |
|  |  |  | OG11843 | Arabinogalactan endo-beta-1,4-galactanase | COG3867 | G |
|  |  |  | OG11859 | Hydrolase | COG0561 | R |
|  |  |  | OG11862 | CRISPR-associated helicase Cas3 | COG1203 | R |
|  |  |  | OG11863 | CRISPR-associated protein, Cse1 family |  |  |
|  |  |  | OG11864 | CRISPR-associated protein |  |  |
|  |  |  | OG11865 | CRISPR-associated protein, Cse4 family |  |  |
|  |  |  | OG11866 | CRISPR-associated protein, Cas5e family |  |  |
|  |  |  | OG11867 | CRISPR-associated protein, Cse3 family |  |  |
|  |  |  | OG11868 | CRISPR-associated endonuclease Cas1 | COG1518 | L |
|  |  |  | OG11869 | 3'-5' Exonuclease with to CRISPR-associated protein from COG1343 | COG2176 | L |
|  |  |  | OG11901 | Prismane protein | COG1073 | R |
|  |  |  | OG11902 | Transposase ORF_A | COG2452 | L |
|  |  |  | OG11909 | Transposase IS607 family protein | COG0675 | L |
|  |  |  | OG11910 | ABC antimicrobial peptide transporter ATPase | COG1136 | V |
|  |  |  | OG11914 | Histidine kinase | COG2972 | T |
|  |  |  | OG11925 | Shikimate dehydrogenase (Fragment) | COG0169 | E |
|  |  |  | OG11949 | Transcriptional regulator, LysR family | COG0583 | K |
|  |  |  | OG11950 | Alpha-galactosidase 1 | COG3345 | G |
|  |  |  | OG11954 | AbpR response regulator | COG3279 | KT |
|  |  |  | OG11962 | Fumarate reductase, flavoprotein subunit, N-terminally truncated | COG1053 | C |
|  |  |  | OG11970 | Pyruvate oxidase | COG0028 | EH |
|  |  |  | OG11990 | Transcriptional regulator, AraC family | COG4753 | T |
|  |  |  | OG11998 | Dinucleotide-utilizing protein | COG0476 | H |
|  |  |  | OG11999 | Transmembrane permease MsmF | COG1175 | G |
|  |  |  | OG12003 | Topology modulation protein | COG0563 | F |
|  |  |  | OG12005 | Cadmium resistance transporter, putative | COG4300 | P |
|  |  |  | OG12008 | FMN-binding domain protein | COG1053 | C |
|  |  |  | OG12010 | Transcriptional regulator SoxR family protein |  |  |
|  |  |  | OG12035 | AbpK sensory transduction histidine kinase | COG2972 | T |
|  |  |  | OG12051 | Putative 5-methylcytosine-specific restriction enzyme subunit McrC | COG4268 | V |
|  |  |  | OG12052 | Glycosyltransferase | COG1216 | R |
|  |  |  | OG12054 | Acyl-CoA dehydrogenase |  |  |
|  |  |  | OG12056 | Substrate-binding protein MsmE | COG1653 | G |
|  |  |  | OG12057 | Rhamnosyltransferase | COG0463 | M |
|  |  |  | OG12099 | Fumarate reductase | COG1053 | C |
|  |  |  | OG12100 | Transcriptional regulator (LysR family) | COG0583 | K |
|  |  |  | OG12101 | R1CVA1 (Transposase) | COG3436 | L |
|  |  |  | OG12118 | Putative TIGR02679 family protein |  |  |
|  |  |  | OG12134 | Diguanylate cyclase (GGDEF) domain protein | COG2199 | T |
|  |  |  | OG12138 | Transposase ORF_A | COG2452 | L |
|  |  |  | OG12139 | Lipase |  |  |
|  |  |  | OG12143 | Branched-chain amino acid transport protein AzlD | COG4392 | S |
|  |  |  | OG12144 | LIV-E family branched chain amino acid exporter AzlC | COG1296 | E |
|  |  |  | OG12159 | FMN-binding domain protein | COG3976 | S |
|  |  |  | OG12225 | Transport protein | COG0477 | GEPR |
|  |  |  | OG12226 | Putative glycosyltransferase WelF | COG1216 | R |
|  |  |  | OG12227 | PTS system, beta-glucoside-specific, IIABC component | COG1263 | G |
|  |  |  | OG12228 | SIS domain protein | COG1737 | K |
|  |  |  | OG12229 | ATPase family associated with various cellular activities (AAA) | COG1401 | V |
|  |  |  | OG12231 | Peptidase, C69 family | COG4690 | E |
|  |  |  | OG12232 | N-acetylmuramoyl-L-alanine amidase |  |  |
|  |  |  | OG12234 | Oligopeptide ABC superfamily ATP binding cassette transporter, binding protein | COG4166 | E |
|  |  |  | OG12235 | Carnosinase | COG0624 | E |
|  |  |  | OG12236 | Bacteriocin helveticin-J |  |  |
|  |  |  | OG12330 | Transposase a | COG1943 | L |
|  |  |  | OG12342 | Transposase | COG0675 | L |
|  |  |  | OG12346 | Major facilitator superfamily MFS_1 transporter (Fragment) |  |  |
|  |  |  | OG12358 | Putative membrane protein |  |  |
|  |  |  | OG12415 | Probable DNA-directed RNA polymerase subunit delta | COG3343 | K |
|  |  |  | OG12437 | Putative glycosyltransferase WelF | COG1216 | R |
|  |  |  | OG12441 | ABC transporter, ATP-binding protein | COG1136 | V |
|  |  |  | OG12443 | LytTr DNA-binding domain protein | COG3279 | KT |
|  |  |  | OG12445 | Oligopeptide binding protein | COG4166 | E |
|  |  |  | OG12448 | PF14243 domain protein |  |  |
|  |  |  | OG12450 | Amidohydrolase | COG1228 | Q |
|  |  |  | OG12451 | Rib/alpha-like repeat protein |  |  |
|  |  |  | OG12453 | ATP-dependent Lon protease | COG4930 | O |
|  |  |  | OG12454 | TIGR02687 family protein |  |  |
|  |  |  | OG12455 | Eco57I restriction endonuclease | COG1002 | V |
|  |  |  | OG12456 | ATPase |  |  |
|  |  |  | OG12458 | Putative inner membrane protein (DUF1819) |  |  |
|  |  |  | OG12460 | AraC-like ligand binding domain protein | COG2207 | K |
|  |  |  | OG12462 | DNA-binding membrane protein | COG1396 | K |
|  |  |  | OG12465 | Glucose-1-phosphate adenylyltransferase, GlgD subunit | COG0448 | G |
|  |  |  | OG13217 | DNA methyltransferase | COG3392 | L |
|  |  |  | OG13218 | DNA adenine methylase (Dam) | COG0338 | L |
|  |  |  | OG13219 | AlwI restriction endonuclease |  |  |
|  |  |  | OG13223 | Transposase IS607 family protein | COG0675 | L |
|  |  |  | OG13224 | Heme ABC transporter, heme-binding protein IsdE | COG0614 | P |
|  |  |  | OG13225 | Ferrichrome ABC transporter, permease | COG0609 | P |
|  |  |  | OG13226 | Iron compound ABC transporter, ATP-binding protein | COG1120 | PH |
|  |  |  | OG13230 | L-Lactate dehydrogenase | COG0039 | C |
|  |  |  | OG13232 | ABC transporter, solute-binding protein | COG2182 | G |
|  |  |  | OG13233 | Putative membrane protein |  |  |
|  |  |  | OG13234 | Oligopeptide ABC superfamily ATP-binding cassette transporter | COG4166 | E |
|  |  |  | OG13235 | Bacteriocin ABC-transporter, ATP-binding and permease component | COG2274 | V |
|  |  |  | OG13236 | Putative bacteriocin |  |  |
|  |  |  | OG13237 | Putative bacteriocin transport accessory protein (Fragment) |  |  |
|  |  |  | OG13242 | Prophage LambdaWp4 DNA methylase | COG1475 | K |
|  |  |  | OG13249 | Oxidoreductase, NAD-binding domain protein | COG0673 | R |
|  |  |  | OG13250 | Ribose 5-phosphate isomerase A | COG0120 | G |
|  |  |  | OG13256 | Rhodanese domain-containing protein |  |  |
|  |  |  | OG13257 | Transposase | COG1943 | L |
|  |  |  | OG13258 | UDP-glucose 4-epimerase |  |  |
|  |  |  | OG13263 | 5-Methyltetrahydropteroyltriglutamate--homocysteine methyltransferase |  |  |
|  |  |  | OG13264 | Transposase ORF_A | COG2452 | L |
|  |  |  | OG13265 | Ser/Thr phosphatase family protein | COG0737 | F |
|  |  |  | OG13267 | Glycosyl hydrolase family 32 | COG1621 | G |
|  |  |  | OG13268 | Transcriptional regulator, LacI family | COG1609 | K |
|  |  |  | OG13270 | Transposase A | COG2452 | L |
|  |  |  | OG13271 | Glycogen synthase | COG0297 | G |
|  |  |  | OG13272 | 1,4-Alpha-glucan branching enzyme | COG0296 | G |
|  |  |  | OG13274 | Fibronectin type III domain protein |  |  |
|  |  |  | OG13276 | Putative DNA-binding protein |  |  |
|  |  |  | OG13279 | UDP-galactopyranose mutase |  |  |
|  |  |  | OG13280 | Undecaprenyl-phosphate galactosephosphotransferase | COG2148 | M |
|  |  |  | OG13281 | Eps11J |  |  |
|  |  |  | OG13282 | NAD dependent epimerase/dehydratase family protein | COG0451 | MG |
|  |  |  | OG13283 | Transposase ORF_A | COG2452 | L |
|  |  |  | OG13286 | Multidrug ABC superfamily ATP binding cassette transporter, ATPase and permease protein | COG2274 | V |
|  |  |  | OG13289 | Death-on-curing family protein | COG3654 | R |
|  |  | Loss | OG10000 | Transposase | COG2826 | L |
|  |  |  | OG10001 | Transposase | COG3547 | L |
|  |  |  | OG10002 | Putative transposase, Mutator family (Fragment) | COG3328 | L |
|  |  |  | OG10003 | Transposase | COG2963 | L |
|  |  |  | OG10004 | Transposase | COG3464 | L |
|  |  |  | OG10007 | Transposase |  |  |
|  |  |  | OG10013 | Putative transposase |  |  |
|  |  |  | OG10015 | ISL3 protein | COG3464 | L |
|  |  |  | OG10176 | Putative cell division protein |  |  |
|  |  |  | OG10978 | Putative phosphorylase (Pnp/Udp family) | COG2820 | F |
|  |  |  | OG11031 | Cation transporting P-type ATPase (Probable copper transporter) | COG2217 | P |
|  |  |  | OG11074 | Polysaccharide biosynthesis protein | COG2244 | R |
|  |  |  | OG11274 | Transposase |  |  |
|  |  |  | OG11345 | Putative pyridine nucleotide-disulphide oxidoreductase | COG1249 | C |
|  |  |  | OG11357 | Dipeptidase | COG4690 | E |
|  |  |  | OG11360 | Transposase |  |  |
|  |  |  | OG11364 | ATP-binding protein involved in Fe/S cluster assembly | COG0396 | O |
|  |  |  | OG11380 | Phosphoenolpyruvate carboxykinase (ATP) |  |  |
|  |  |  | OG11411 | Transport permease protein | COG0842 | V |
|  |  |  | OG11412 | ABC transporter, ATP-binding protein | COG1131 | V |
|  |  |  | OG11427 | Shikimate kinase | COG0703 | E |
|  |  |  | OG11432 | Fe-S cluster assembly ABC-type transport system, permease component | COG0719 | O |
|  |  |  | OG11437 | LPXTG-motif cell wall anchor domain protein |  |  |
|  |  |  | OG11438 | Amino acid ABC transporter substrate-binding protein, PAAT family | COG0834 | ET |
|  |  |  | OG11439 | Amino acid ABC transporter ATP-binding protein, PAAT family | COG1126 | E |
|  |  |  | OG11440 | Amino acid ABC transporter membrane protein, PAAT family | COG0765 | E |
|  |  |  | OG11442 | Transposase |  |  |
|  |  |  | OG11451 | Transposase |  |  |
|  |  |  | OG11506 | Two-component system, response regulator | COG3279 | KT |
|  |  |  | OG11559 | Transposase | COG3335 | L |
|  |  |  | OG11592 | Putative membrane protein |  |  |
|  |  |  | OG11627 | Transposase | COG2963 | L |
|  |  |  | OG11638 | Oligopeptide binding protein | COG4166 | E |
|  |  |  | OG11717 | Oligopeptide ABC transporter substrate-binding protein | COG4166 | E |
|  |  |  | OG11813 | Haloacid dehalogenase | COG0561 | R |

**Supplementary table 9. Orthologous genes gained/lost in the *indicus* lineage.**

| 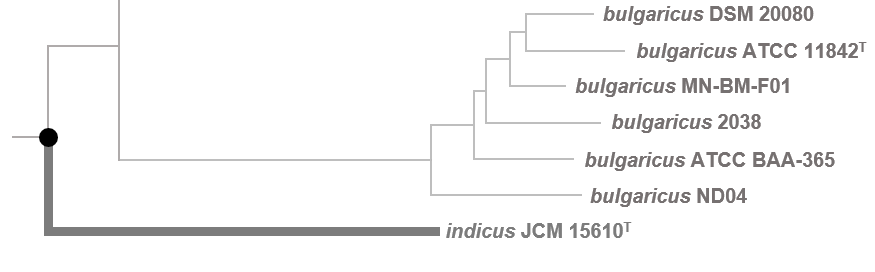 | | | | | | |
| --- | --- | --- | --- | --- | --- | --- |
| **Evolution path** | | **Gain/**  **loss** | **Orthologous gene_Fam** | **Annotation** | **COG No.** | **COG cat.** |
| **From** | **To** |  |  |  |  |  |
| Common ancestor of *Lactobacillus delbrueckii* | *indicus* lineage | Gain | OG10003 | Transposase | COG2963 | L |
|  |  |  | OG10004 | Transposase | COG3464 | L |
|  |  |  | OG10010 | Transposase ORF_B | COG0675 | L |
|  |  |  | OG10011 | Degenerate transposase | COG3436 | L |
|  |  |  | OG10014 | Transposase, IS4 family | COG3385 | L |
|  |  |  | OG10015 | ISL3 protein | COG3464 | L |
|  |  |  | OG10033 | 1-Deoxy-D-xylulose 5-phosphate synthase | COG1154 | HI |
|  |  |  | OG10080 | Hydrolase acting on acid anhydrides, catalyzing transmembrane movement of substances | COG0474 | P |
|  |  |  | OG10083 | Phosphoglycerate dehydrogenase | COG0111 | HE |
|  |  |  | OG10120 | Integrase/recombinase | COG0582 | L |
|  |  |  | OG10121 | Glycerate kinase | COG1929 | G |
|  |  |  | OG10161 | Dihydroorotase | COG0044 | F |
|  |  |  | OG10163 | Putative Transposase | COG0675 | L |
|  |  |  | OG10619 | ABC-type proline/glycine betaine transport system, permease and substrate binding protein | COG1732 | M |
|  |  |  | OG11038 | Cystathionine gamma-lyase | COG0626 | E |
|  |  |  | OG11054 | ABC transporter, ATP-binding protein | COG1122 | P |
|  |  |  | OG11183 | Type I restriction-modification system, restriction subunit | COG0610 | V |
|  |  |  | OG11186 | Putative acetyltransferase | COG2388 | R |
|  |  |  | OG11281 | ABC transporter, ATP-binding/permease protein | COG1132 | V |
|  |  |  | OG11302 | Rhodanese-related sulfurtransferase | COG0607 | P |
|  |  |  | OG11303 | Putative phosphoglycolate phosphatase | COG0546 | R |
|  |  |  | OG11348 | Lactate dehydrogenase related enzyme | COG0111 | HE |
|  |  |  | OG11350 | ABC transporter, ATP-binding/permease protein | COG1132 | V |
|  |  |  | OG11351 | ABC transporter, ATP-binding/permease protein | COG1132 | V |
|  |  |  | OG11374 | LysR family transcriptional regulator | COG0583 | K |
|  |  |  | OG11401 | Transposase family protein A | COG1943 | L |
|  |  |  | OG11418 | Putative transcriptional regulator (LysR family) | COG0583 | K |
|  |  |  | OG11450 | Bacterial membrane protein yfho |  |  |
|  |  |  | OG11453 | Transposase |  |  |
|  |  |  | OG11462 | Ribonuclease H | COG0328 | L |
|  |  |  | OG11480 | Putative signal transduction protein | COG2199 | T |
|  |  |  | OG11514 | Protein kinase, putative | COG0515 | RTKL |
|  |  |  | OG11518 | Integrase/recombinase | COG0582 | L |
|  |  |  | OG11552 | Transposase | COG3464 | L |
|  |  |  | OG11574 | Transposase | COG3547 | L |
|  |  |  | OG11575 | Type I restriction-modification system, modification subunit | COG0286 | V |
|  |  |  | OG11586 | ABC transporter, substrate-binding protein | COG2984 | R |
|  |  |  | OG11588 | Spermidine/putrescine ABC transporter, ATP-binding protein | COG3842 | E |
|  |  |  | OG11599 | ABC transporter ATP-binding protein |  |  |
|  |  |  | OG11611 | Type III restriction enzyme, res subunit | COG0827 | L |
|  |  |  | OG11615 | PTS family fructose/mannitol porter, component IIA | COG1762 | GT |
|  |  |  | OG11622 | Type I restriction-modification system, specificity subunit | COG0732 | V |
|  |  |  | OG11627 | Transposase | COG2963 | L |
|  |  |  | OG11629 | EpsIIB, Putative glycosyltransferase |  |  |
|  |  |  | OG11640 | Succinate dehydrogenase | COG1053 | C |
|  |  |  | OG11660 | Transcription antiterminator LacT | COG3711 | K |
|  |  |  | OG11661 | PTS family lactose porter, IICB component | COG1455 | G |
|  |  |  | OG11673 | Transcriptional regulator |  |  |
|  |  |  | OG11699 | Spermidine/putrescine ABC transporter, substrate binding protein | COG0687 | E |
|  |  |  | OG11716 | Galactose-6-phosphate isomerase LacB subunit | COG0698 | G |
|  |  |  | OG11720 | Phosphoenolpyruvate-dependent sugar phosphotransferase system EIIA, lactose specific | COG1447 | G |
|  |  |  | OG11721 | PTS family galactitol (Gat) porter, component IIC | COG3775 | G |
|  |  |  | OG11726 | Oligopeptide ABC superfamily ATP binding cassette transporter | COG4166 | E |
|  |  |  | OG11740 | Acyl-CoA dehydrogenase |  |  |
|  |  |  | OG11757 | Putative ABC transporter, ATP-binding protein | COG1116 | P |
|  |  |  | OG11771 | 6-Phospho-beta-glucosidase / 6-phospho-beta-galactosidase | COG2723 | G |
|  |  |  | OG11779 | Transposase |  |  |
|  |  |  | OG11785 | Putative glucose-1-phosphate thymidylyltransferase | COG1209 | M |
|  |  |  | OG11809 | Extracellular zinc metalloproteinase |  |  |
|  |  |  | OG11810 | ABC superfamily ATP binding cassette transporter, permease | COG0395 | G |
|  |  |  | OG11820 | Galactose-6-phosphate isomerase subunit LacA | COG0698 | G |
|  |  |  | OG11822 | XRE family transcriptional regulator |  |  |
|  |  |  | OG11826 | 6-Phospho-beta-galactosidase | COG2723 | G |
|  |  |  | OG11827 | Membrane-bound protein |  |  |
|  |  |  | OG11828 | PTS system, galacitol-specific IIB component | COG3414 | G |
|  |  |  | OG11830 | Tagatose 1,6-diphosphate aldolase | COG3684 | G |
|  |  |  | OG11831 | Tagatose-6-phosphate kinase | COG1105 | G |
|  |  |  | OG11832 | Aldose epimerase | COG2017 | G |
|  |  |  | OG11833 | Conserved protein |  |  |
|  |  |  | OG11841 | DNA helicase | COG1112 | L |
|  |  |  | OG11842 | Transcription regulator | COG0583 | K |
|  |  |  | OG11859 | Hydrolase | COG0561 | R |
|  |  |  | OG11862 | CRISPR-associated helicase Cas3 | COG1203 | R |
|  |  |  | OG11863 | CRISPR-associated protein, Cse1 family |  |  |
|  |  |  | OG11864 | CRISPR-associated protein |  |  |
|  |  |  | OG11865 | CRISPR-associated protein, Cse4 family |  |  |
|  |  |  | OG11866 | CRISPR-associated protein, Cas5e family |  |  |
|  |  |  | OG11867 | CRISPR-associated protein, Cse3 family |  |  |
|  |  |  | OG11868 | CRISPR-associated endonuclease Cas1 | COG1518 | L |
|  |  |  | OG11869 | 3'-5' Exonuclease with to CRISPR-associated protein from COG1343 | COG2176 | L |
|  |  |  | OG11871 | Putative type-I specificity determinant subunit | COG0732 | V |
|  |  |  | OG11902 | Transposase ORF_A | COG2452 | L |
|  |  |  | OG11909 | Transposase IS607 family protein | COG0675 | L |
|  |  |  | OG11911 | Sugar ABC transporter permease protein | COG1175 | G |
|  |  |  | OG11912 | Metallo-beta-lactamase superfamily hydrolase | COG0595 | R |
|  |  |  | OG11913 | ABC superfamily ATP binding cassette transporter | COG1653 | G |
|  |  |  | OG11931 | ROK family protein | COG1940 | KG |
|  |  |  | OG11936 | MerR family transcription regulator | COG0789 | K |
|  |  |  | OG11942 | Stress response regulator Gls24 | COG1302 | S |
|  |  |  | OG11969 | Putative transposase |  |  |
|  |  |  | OG11975 | EpsIM, Putative glycosyltransferase | COG0438 | M |
|  |  |  | OG11978 | Spermidine/putrescine ABC transporter, permease protein | COG1176 | E |
|  |  |  | OG11979 | Spermidine/putrescine ABC transporter, permease protein | COG1177 | E |
|  |  |  | OG11980 | Adenine deaminase | COG1001 | F |
|  |  |  | OG11989 | Shikimate 5-dehydrogenase | COG3976 | S |
|  |  |  | OG11990 | Transcriptional regulator, AraC family | COG4753 | T |
|  |  |  | OG12004 | 3-Beta hydroxysteroid dehydrogenase | COG0702 | MG |
|  |  |  | OG12008 | FMN-binding domain protein | COG1053 | C |
|  |  |  | OG12012 | Type I site-specific deoxyribonuclease | COG0732 | V |
|  |  |  | OG12037 | Type I restriction-modification system specificity subunit |  |  |
|  |  |  | OG12045 | Probable membrane protein | COG3274 | S |
|  |  |  | OG12048 | Cell surface protein (Fragment) |  |  |
|  |  |  | OG12049 | FAD binding domain protein | COG1053 | C |
|  |  |  | OG12050 | Chromosome partitioning ATPase | COG1192 | D |
|  |  |  | OG12051 | Putative 5-methylcytosine-specific restriction enzyme subunit McrC | COG4268 | V |
|  |  |  | OG12052 | Glycosyltransferase | COG1216 | R |
|  |  |  | OG12085 | Putative transcriptional regulator | COG2865 | K |
|  |  |  | OG12094 | 1-Deoxy-D-xylulose 5-phosphate synthase | COG1154 | HI |
|  |  |  | OG12098 | Transferase for other substituted phosphate groups | COG3475 | M |
|  |  |  | OG12104 | Adenine deaminase | COG1001 | F |
|  |  |  | OG12130 | Transposase | COG2826 | L |
|  |  |  | OG12134 | Diguanylate cyclase (GGDEF) domain protein | COG2199 | T |
|  |  |  | OG12146 | Major facilitator superfamily permease |  |  |
|  |  |  | OG12147 | Transporter protein | COG0477 | GEPR |
|  |  |  | OG12148 | HesA/MoeB/ThiF family protein | COG0476 | H |
|  |  |  | OG12151 | Transcriptional regulator | COG1846 | K |
|  |  |  | OG12152 | ABC transporter, ATP-binding protein | COG1116 | P |
|  |  |  | OG12165 | Type I restriction-modification system DNA-methyltransferase | COG0286 | V |
|  |  |  | OG12170 | Transposase IS4 family protein | COG3293 | L |
|  |  |  | OG12174 | Transposase |  |  |
|  |  |  | OG12211 | Periplasmic protease | COG0791 | M |
|  |  |  | OG12215 | Diguanylate cyclase domain protein |  |  |
|  |  |  | OG12216 | Amino-acid transporter | COG0531 | E |
|  |  |  | OG12217 | Insertion element | COG2826 | L |
|  |  |  | OG12219 | Transport protein | COG2252 | R |
|  |  |  | OG12220 | Adenine deaminase | COG1816 | F |
|  |  |  | OG12221 | MATE efflux family protein | COG0534 | V |
|  |  |  | OG12223 | Type I restriction-modification system, S subunit | COG0732 | V |
|  |  |  | OG12225 | Transport protein | COG0477 | GEPR |
|  |  |  | OG12226 | Putative glycosyltransferase WelF | COG1216 | R |
|  |  |  | OG12355 | Group 2 glycosyl transferase | COG3754 | M |
|  |  |  | OG12383 | Helicase conserved C-terminal domain protein | COG0514 | L |
|  |  |  | OG12385 | Transposase ORF_A | COG2452 | L |
|  |  |  | OG12399 | Transposase family protein A | COG1943 | L |
|  |  |  | OG12409 | Transposase, IS30 family | COG2826 | L |
|  |  |  | OG12415 | Probable DNA-directed RNA polymerase subunit delta | COG3343 | K |
|  |  |  | OG12416 | Lipoprotein, peptide binding protein OppA-like protein | COG4166 | E |
|  |  |  | OG12420 | Transcriptional regulator, MerR family |  |  |
|  |  |  | OG12421 | Metal ion ABC superfamily ATP binding cassette transporter, membrane protein |  |  |
|  |  |  | OG12422 | Cobalt transport protein | COG0619 | P |
|  |  |  | OG12423 | Transposase for insertion sequence element | COG3335 | L |
|  |  |  | OG12426 | Transcription regulator |  |  |
|  |  |  | OG12429 | Putative transposase DNA-binding domain protein | COG0675 | L |
|  |  |  | OG12433 | Sugar kinase, ribokinase family | COG0524 | G |
|  |  |  | OG12436 | Putative glycosyl transferase | COG0438 | M |
|  |  |  | OG12438 | Transposase | COG0675 | L |
|  |  |  | OG13093 | Cobalt ABC transporter, ATP-binding protein (Putative) | COG1122 | P |
|  |  |  | OG13094 | Nickel ECF transporter,substrate-specific component NikM | COG0310 | P |
|  |  |  | OG13095 | Crp/FNR family transcriptional regulator | COG0664 | T |
|  |  |  | OG13096 | Lactate racemization operon protein LarA | COG3875 | S |
|  |  |  | OG13097 | Lactate racemization operon protein LarB | COG1691 | R |
|  |  |  | OG13098 | Lactate racemization operon protein LarC, N-terminal domain protein | COG1641 | S |
|  |  |  | OG13099 | Lactate racemization operon protein | COG1641 | S |
|  |  |  | OG13100 | Glycerol transporter | COG0580 | G |
|  |  |  | OG13101 | Lactate racemization operon protein LarE | COG1606 | R |
|  |  |  | OG13104 | Transposase | COG2826 | L |
|  |  |  | OG13105 | Transposase | COG2826 | L |
|  |  |  | OG13106 | Putative permease | COG0471 | P |
|  |  |  | OG13108 | Putative DNA methyltransferase yeeA | COG1002 | V |
|  |  |  | OG13109 | Putative ATP-dependent helicase yeeB |  |  |
|  |  |  | OG13111 | Transposase |  |  |
|  |  |  | OG13112 | Transposase for insertion sequence element |  |  |
|  |  |  | OG13113 | Oligopeptide ABC superfamily ATP binding cassette transporter, binding protein | COG4166 | E |
|  |  |  | OG13114 | Oligopeptide ABC superfamily ATP binding cassette transporter, binding protein | COG4166 | E |
|  |  |  | OG13118 | Transposase | COG0675 | L |
|  |  |  | OG13119 | Transposase |  |  |
|  |  |  | OG13121 | Transposase ORF_A | COG2452 | L |
|  |  |  | OG13122 | Transposase |  |  |
|  |  |  | OG13124 | Sortase | COG3764 | M |
|  |  |  | OG13127 | Possible transposase |  |  |
|  |  |  | OG13128 | Prephenate dehydrogenase | COG0287 | E |
|  |  |  | OG13130 | Nicotinate-nucleotide pyrophosphorylase | COG0157 | H |
|  |  |  | OG13131 | Major facilitator transporter | COG0477 | GEPR |
|  |  |  | OG13132 | Transposase | COG3464 | L |
|  |  |  | OG13133 | Homoserine O-succinyltransferase | COG1897 | E |
|  |  |  | OG13140 | Transposase, ISSmi4 | COG3436 | L |
|  |  |  | OG13143 | Transposase, IS30 family | COG2826 | L |
|  |  |  | OG13144 | Transposase, IS30 family | COG2826 | L |
|  |  |  | OG13146 | Transposase, IS30 family | COG2826 | L |
|  |  |  | OG13147 | Endopeptidase | COG3590 | O |
|  |  |  | OG13150 | Restriction endonuclease EcoKI subunit R | COG4096 | V |
|  |  |  | OG13151 | AAA domain protein | COG1112 | L |
|  |  |  | OG13152 | AAA domain protein | COG1112 | L |
|  |  |  | OG13157 | ABC transporter ATP-binding protein | COG1136 | V |
|  |  |  | OG13170 | Peptide binding protein | COG4166 | E |
|  |  |  | OG13173 | Sugar kinase, ribokinase family |  |  |
|  |  |  | OG13174 | Transposase | COG2826 | L |
|  |  |  | OG13178 | NAD dependent epimerase/dehydratase family protein | COG0451 | MG |
|  |  |  | OG13179 | 4-Diphosphocytidyl-2C-methyl-D-erythritol synthase | COG1211 | I |
|  |  |  | OG13180 | Glycosyltransferase, group 2 family protein | COG0463 | M |
|  |  |  | OG13186 | DNA methyltransferase | COG0338 | L |
|  |  |  | OG13189 | Glycosyltransferase group 2 family protein |  |  |
|  |  |  | OG13190 | NAD dependent epimerase/dehydratase family protein | COG0451 | MG |
|  |  |  | OG13191 | Acetyl transferase | COG0110 | R |
|  |  |  | OG13192 | Wzy |  |  |
|  |  |  | OG13193 | Transposase, partial | COG2452 | L |
|  |  |  | OG13200 | ABC superfamily ATP binding cassette transporter | COG1653 | G |
|  |  |  | OG13201 | ABC transporter, solute-binding protein | COG1653 | G |
|  |  |  | OG13202 | Metallo-beta-lactamase superfamily hydrolase | COG0595 | R |
|  |  |  | OG13204 | ABC superfamily ATP binding cassette transporter, permease protein | COG0395 | G |
|  |  |  | OG13205 | Sugar ABC transporter permease protein | COG1175 | G |
|  |  |  | OG13206 | FAD binding domain protein |  |  |
|  |  |  | OG13207 | Predicted phosphatase | COG0546 | R |
|  |  |  | OG13209 | Transcription regulator | COG0583 | K |
|  |  | Loss | - | - | - | - |

**Supplementary figure 1. Average genome size of *Lactobacillus delbrueckii* at the subspecies level.**


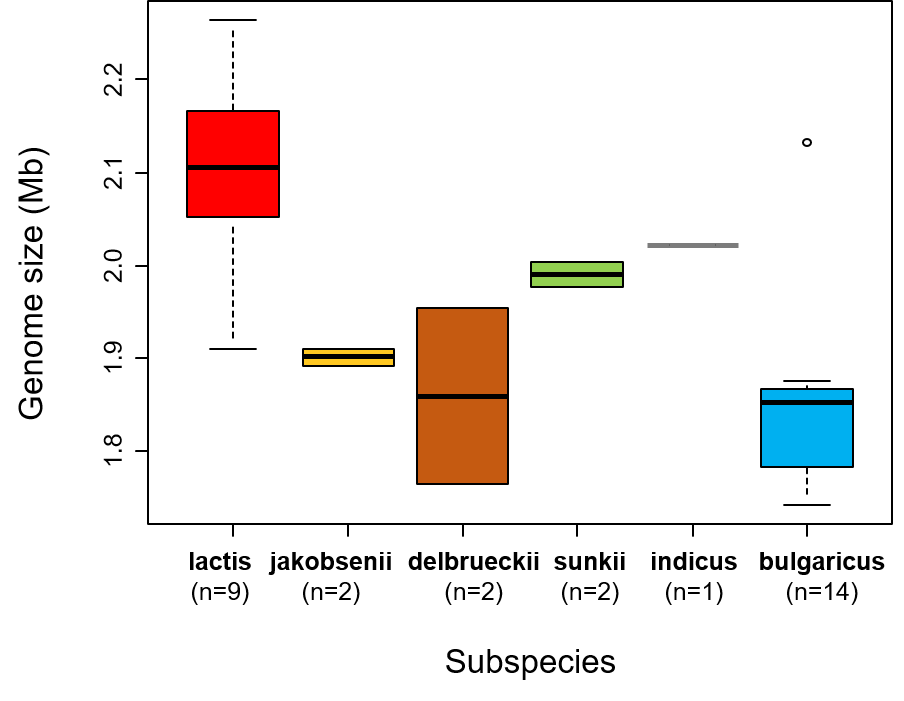


**Supplementary figure 2. The pan- and core-genome size prediction of 31 *L. delbrueckii* strains**. (A) The graphs represent the number of new genes (blue boxes) and the core gene count (red boxes) after a sequential addition of each genome to the analysis. The number of core orthologous genes of 31 genomes is 1,069. The pan-genome of 31 genomes includes 4,332 orthologous genes. (B) New gene family distribution after a sequential addition of each genome to the analysis.


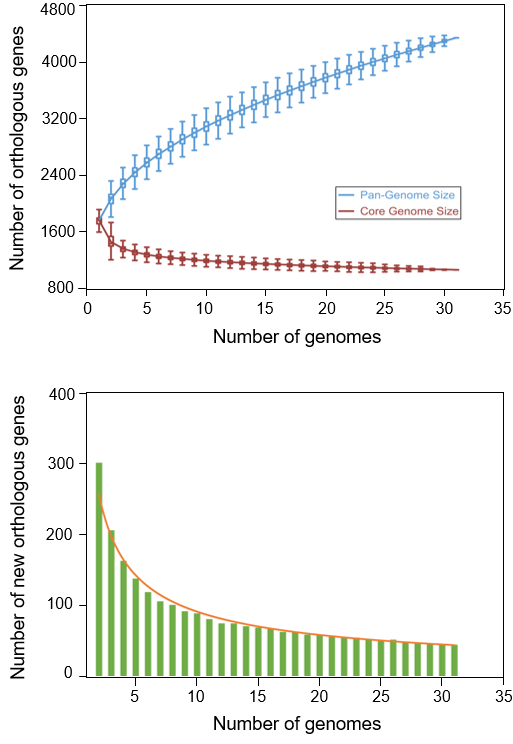

Supplement: Supplementary file 3 — Supplementary Information 3. [file 41598_2023_29404_MOESM3_ESM.docx]
